# *MALAT1* as master regulator of biomarkers predictive of pan-cancer multi-drug resistance in the context of recalcitrant NRAS signaling pathway identified using systems-oriented approach

**DOI:** 10.1038/s41598-022-11214-8

**Published:** 2022-05-09

**Authors:** Santosh Kumar, Seema Mishra

**Affiliations:** Department of Biochemistry, School of Life Sciences, University of Hyderabad, Hyderabad, Telangana 500046 India

**Keywords:** Systems biology, Regulatory networks, Systems analysis, Computational biology and bioinformatics, Cellular signalling networks, Functional clustering, Gene regulatory networks, Genome informatics, Microarrays

## Abstract

NRAS, a protein mutated in several cancer types, is involved in key drug resistance mechanisms and is an intractable target. The development of drug resistance is one of the major impediments in targeted therapy. Currently, gene expression data is used as the most predictive molecular profile in pan-cancer drug sensitivity and resistance studies. However, the common regulatory mechanisms that drive drug sensitivity/resistance across cancer types are as yet, not fully understood. We focused on GDSC data on NRAS-mutant pan-cancer cell lines, to pinpoint key signaling targets in direct or indirect associations with NRAS, in order to identify other druggable targets involved in drug resistance. Large-scale gene expression, comparative gene co-expression and protein–protein interaction network analyses were performed on selected drugs inducing drug sensitivity/resistance. We validated our data from cell lines with those obtained from primary tissues from TCGA. From our big data studies validated with independent datasets, protein-coding hub genes *FN1, CD44, TIMP1, SNAI2,* and *SPARC* were found significantly enriched in signal transduction, proteolysis, cell adhesion and proteoglycans pathways in cancer as well as the PI3K/Akt-signaling pathway*.* Further studies of the regulation of these hub/driver genes by lncRNAs revealed several lncRNAs as prominent regulators, with *MALAT1* as a possible master regulator. Transcription factor EGR1 may control the transcription rate of *MALAT1* transcript. Synergizing these studies, we zeroed in on a pan-cancer regulatory axis comprising EGR1-*MALAT1*-driver coding genes playing a role. These identified gene regulators are bound to provide new paradigms in pan-cancer targeted therapy, a foundation for precision medicine, through the targeting of these key driver genes in the improvement of multi-drug sensitivity or resistance.

## Introduction

Cancer is a serious health issue and the second leading cause of death worldwide as estimated by World Health Organization^1^. Drug resistance which can be acquired or intrinsic, develops due to the failure of chemotherapeutic drugs to treat cancer cells because of limited effectiveness^2–4^. While intrinsic antibiotic/drug resistance is a naturally occurring phenomenon primarily present before chemotherapy^5,6^, acquired drug resistance arises after the chemotherapeutic treatment of cancer^2^.

Intrinsic drug resistance may arise due to existential mutations in crucial genes, intrinsic heterogeneity of tumors, and/or activation of certain molecular pathways against anti-cancer drugs^6^. In one study, transcriptional repressors Snail and Slug were observed to induce radioresistance and chemoresistance in ovarian cancer through the antagonism of p53-mediated apoptosis^7^. Acquired drug resistance may be the result of activation of secondary proto-oncogenes, mutations or altered expression of drug targets and post-treatment changes in the tumor microenvironment. Reiterating, there are several possible mechanisms involved in cancer drug resistance, including altered expression and mutation in target oncogenes, compensatory activation of the downstream signaling pathways, epigenetic abnormalities and histological transformations^8,9^. Drug resistance can also occur due to alterations in the molecular structures of drug targets. As an example, imatinib resistance was reported due to gatekeeper mutation in the oncogenic kinase domain of BCR-ABL1 (T315) in chronic myeloid leukemia (CML) patients^2^. In one study, tumors were observed to develop enzalutamide-resistance through lineage plasticity^10^. ABC transporter family proteins have also been found to be responsible for multi-drug resistance in several tumors, with MDR1/P-glycoprotein or ABCB1 as one of the first factors identified in an in vitro model^11^. In order to overcome drug resistance, several cancer genomic biomarkers have been identified, which are highly associated with anti-cancer drug sensitivity in cancer cell lines^12^. Many new anti-cancer drugs have been screened at a large scale against a wide range of human cancer cell lines to uncover clinically meaningful gene-drug interactions^13,14^. Such studies on gene/protein-drug interactions are important in identifying and sorting the problem of drug resistance and in proposing novel therapeutic biomarkers.

An intensive search for the key genes frequently involved in cancer drug resistance led us to the RAS-RAF family of genes. Kinases encoded by the RAF gene family which play an important role in cell growth, proliferation and differentiation are regulated by RAS. Mutations in the RAS family of proteins can influence phenotypes. Several novel roles of this family of proteins in human genetic disorders have been proposed^15^. Studies suggest that mutations in *NRAS* and *BRAF* are associated with the declining survival rate of metastatic cancer patients^16^. In one study^17^, however, mutant NRAS was not found to be significantly associated with survival in colon cancer patients. It is suggested that NRAS mutations might arise under the conditions of chronic apoptotic stress^18^, and that the mutation of NRAS may suppress apoptosis^18,19^. Mutation in NRAS is associated with the mechanisms involved in drug resistance^20,21^. In the former study, it was found that in mutant BRAF melanoma cells with acquired vemurafenib-resistance mediated by secondary mutation in NRAS, PB04 inhibited the phosphorylation of ERK1/2, while in the latter study, it was observed that the acquired resistance to B-RAF inhibitor developed through NRAS mutations. RAS-targeted therapy has long been elusive^22^. Mutant NRAS is constitutively active, and very difficult to target directly. Hence, the development of drugs for NRAS is largely unsuccessful. Several strategies for targeting are beginning to be explored^23^. MEK inhibitor binimetinib was used in a phase III trial to assess the efficacy and safety against NRAS-mutant melanoma, however, data is as yet insufficient to reach a definite conclusion as to its efficacy improvement^24–26^. Phase 1 clinical trials of LXH254, a pan-RAF inhibitor, in patients with solid tumors harboring MAPK pathway alterations have just been completed in February 2022 (https://clinicaltrials.gov/ct2/show/NCT02607813), and final results remain to be disseminated. Currently, to the best of our knowledge, there is no targeted therapy that has yet been approved for *NRAS*-mutant cancer, although several inhibitors are under investigation^26,27^.

It is widely known that bound RAS proteins activate several downstream pathways and, in this manner, can act as an effector^28^. In order to screen for druggable targets*,* which may be in direct or indirect association with *NRAS*, we hypothesized that such genes other than *NRAS* present in the MAPK signaling pathway, may be promising targets. Further, understanding the regulatory environment of such targets will be key to circumvent the effects of refractory mutant *NRAS* in drug resistance. Towards this, in addition to the omnipresent proteins as regulators, we focused on the identification of key long non-coding RNAs (lncRNAs), the newly emerging ones, to pinpoint key master regulators of select coding genes. LncRNAs through their actions on such predictive biomarker targets may directly or indirectly regulate pan-cancer drug sensitivity and resistance.

LncRNAs alter gene expression in a variety of cancer types^29,30,31^. Saleembhasha and Mishra ^32^, first published in 2017 working with a TCGA RNA-seq dataset of 5601 samples from 15 different primary cancer types, proposed a pan-cancer regulatory axis consisting of *PVT1*, E2F1 and FOXM1 as common gene expression regulatory entity. Other lncRNAs have also been implicated to function as master regulators of overexpressed common coding genes involved in primary pan-cancer development and among these, *PVT1, SNHG11* and *MIR22HG* are deduced to be key regulatory lncRNAs^32^. Further, it has been shown that lncRNAs have a significant impact on the cancer drug resistance in many cancer types^29,33^. As an example, *TP73-AS1* lncRNA was found to induce temazolamide (TZM) resistance in glioblastoma cancer stem cells by altering ALDH1A1 expression^34^ and *HOTAIR1* lncRNA was shown to promote tamoxifen resistance in breast cancer by activating estrogen receptor (ER) signaling^35^. However, the exact molecular mechanisms of several lncRNAs involved in cancer drug resistance have not been fully characterized.

In this study, we probed the likely functional roles of predictive biomarkers and their regulatory mechanisms in pan-cancer drug resistance by employing microarray data and drug response data from the updated Genomics of Drug Sensitivity in Cancer (GDSC) database. We validated our observations on significantly differentially expressed genes (DEGs) obtained from cell lines with those obtained from cancer primary tissues from patients, which is deposited in TCGA. We also constructed gene co-expression, protein–protein interaction and regulatory networks and analyzed the networks both qualitatively and quantitatively^36^ to pinpoint probable biomarkers. Further, comprehensive studies on the regulation of these druggable targets by lncRNAs at the mRNA level, provided novel insights into their regulatory pattern and mechanisms. These insights are expected to help improve pan-cancer drug sensitivity to these select drugs acting on the molecules involved in the *NRAS* signaling pathway, and will also be useful in drug repurposing studies utilizing our chosen targets.

## Materials and methods

### Drug response data analysis taken from genomics of drug sensitivity in cancer (GDSC) database

GDSC database is the largest publicly available resource for information on drug sensitivity in cancer cells and molecular markers of drug response, gene expression, and copy number alteration for thousands of cancer cell lines. Currently, it contains approximately 2,12,774 drug dose–response measurements for drug sensitivity/resistance. It harbors data for about 265 drugs with their sensitivities measured across thousands of cancer cell lines derived from primary cells of different tissues including cancers of lung (179 cell lines), hematopoietic & lymphoid tissues (175 cell lines), skin (62 cell lines), CNS (58 cell lines) and breast (52 cell lines), among others. (Supplementary Fig. S1a)^13,37^. These screened anti-cancer drugs include clinical drugs (n = 48), drugs in clinical development (n = 76), and experimental compounds (n = 141), targeting a wide range of biomarkers and biological pathways such as apoptosis, transcription regulation, DNA repair and protein kinase pathways. (Supplementary Fig. S1b).

### Cancer cell lines harboring mutant NRAS: drug sensitivity data

For cell lines harboring mutant *NRAS* gene, analysis of variance (ANOVA) test to uncover the gene-drug associations for drug sensitivity and resistance in cancer cell lines based on drug IC_50_ value, has been performed and deposited in GDSC itself. From this ANOVA analysis on *NRAS*-mutant vs *NRAS*-wild type cancer cell lines, 12 drugs were found to be significantly associated (threshold *p* < 0.0001, FDR ≤ 25%) with either drug sensitivity or resistance (https://www.cancerrxgene.org; Supplementary Fig. S2) and therefore, were enlisted. In *NRAS*-mutant cell lines, drug sensitivity was attributed to MEK1/2, BRAF, TAK and MAP4K2 inhibitors (*p*-values = 3.04 × 10^–10^ for PD0325901, 3.38 × 10^–4^ for PLX4720, 1.05 × 10^–5^ for TL-1–85 and 1.55 × 10^–5^ for NG-25), respectively. However, *NRAS*-mutant cancer cell lines were significantly resistant to Foretinib, a MET inhibitor (*p*-value = 2.61 × 10^–4^) and also resistant to Ponatinib (*p*-value = 2.99 × 10^–5^) and Cabozantinib (*p*-value = 1.61 × 10^–4^), which are multiple target inhibitors (Supplementary Table S1). A schematic representation of the methods used is depicted as a flow chart (Supplementary Fig. S3a).

### Drug sensitivity data

We selected only those *NRAS*-mutant cancer cell lines that were commonly responsive to all 10 drugs. We downloaded IC_50_ (log normalized) drug sensitivity data for these 10 drugs across *NRAS*-mutant (total 41) cancer cell lines from different tissue types^13^. We excluded RDEA119 (Refametinib), also seen involved, from our further analysis due to contradictory drug LN_IC_50_ values deposited twice in GDSC. GDSC database suggested that the cancer cell lines with an LN_IC_50_ (Log Normalized Half-maximal Inhibitory Concentration) value greater than the maximum concentration of a drug are considered as drug-resistant cell lines, whereas cell lines with LN_IC_50_ value smaller than the maximum concentration of a drug are considered as a drug-sensitive cell line. We generated a clustered heatmap (unsupervised clustering) with uncentered correlation and average linkage using LN_IC_50_ values *vs* cancer cell lines in online tool GenePattern v11 and visualized it in TreeView version 1.1.

### Validation with tissue samples

To correlate the drug sensitivity and resistance of cancer cell lines with that of cancer tissues for the same drugs as a means for independent validation, we downloaded the predicted drug IC_50_ value of 10 drugs for the *NRAS*-mutant cancer tissue from the database CancerRxTissue (https://manticore.niehs.nih.gov/cancerRxTissue), which extracts molecular data from patient samples deposited in TCGA to predict drug sensitivity, for the same cancer types as represented by the studied cell lines. *NRAS* and related genes mutation information was obtained from the TCGA database (https://www.cancer.gov/about-nci/organization/ccg/research/structural-genomics/tcga). We generated a heatmap using the above method as used for cancer cell lines.

### Significant differential gene expression analysis

In order to study significant differential gene expression in cancer cell lines, we downloaded basal gene expression profile data from the GDSC database and filtered the expression value of missing gene names from the column and performed an unpaired t-test using Multi Experiment Viewer (MeV) version 4.9.0 with unequal group variance (Welch approximation) between two subjects (drug-sensitive and -resistant cancer cell lines), with threshold cut off *p*-value < 0.05. We analyzed basal gene expression profile data of 17,417 genes in each of the *NRAS*-mutant cell lines which were generated using the Affymetrix Human Genome U219 Array. From our heatmap based on IC_50_ values, a total of 44 drug-sensitive and 68 drug-resistant cell lines belonging to 5 out of 10 drugs were analyzed. In the case of the remaining 5 of these drugs, we were not able to clearly classify samples into drug-resistant/sensitive classes. For our analysis, we took gene expression data of cell lines selected as above, which was normalized using a robust multi-array average (RMA) algorithm. A volcano plot to visualize up- and down-regulated genes between the two groups using identified differentially expressed genes (DEGs) via the R package “ggplot2” with double filtration cut off *p*-value < 0.05, and logFC > 2 was also generated. To identify the names of these specific genes, and also visualize the pattern of gene expression in each drug-sensitive and resistant cell line, we further generated a heatmap using Comparative Marker Selection in Gene Pattern version 11 (https://cloud.genepattern.org) with default parameters.

To identify common genes differentially expressed across the multiple drugs, we used online web tool BioInfoRX (http://apps.bioinforx.com/bxaf6/tools) to generate a Venn diagram. Further, a bubble plot was generated to visualize these DEGs overlapping across multiple drugs (at least for three drugs) using the R package “ggplot2 & ggpubr”.

### Functional gene enrichment annotation analysis

We performed functional gene enrichment analysis to find out the functional implications of DEGs in drug-resistant/sensitive cancer cell lines. This was carried out in the context of 5 drugs using GeneCodis (Gene annotations co-occurrence discovery) version 4 web-accessible bioinformatics tool (http://genecodis.cnb.csic.es/analysis). For a significant enrichment of genes, the threshold hypergeometric (defined as the Benjamini-adjusted Fisher’s exact test *p*-value) *p*-value < 0.05 (default) was used.

### Construction and analysis of gene co-expression network

In order to study the molecular interaction of these genes, we constructed a co-expression network. Using DEGs between drug-resistant and sensitive cancer cell lines, we submitted the list of DEGs set into an extensively validated online web server GeneMANIA program (https://genemania.org). It provides a significant gene–gene interaction network between DEGs, and also some additional genes from GeneMANIA are added by default, if found to be interacting with the submitted gene list.

A node having a higher number of edges with interacting nodes is considered a key/hub gene node. The co-expression network was analyzed to detect hub genes by using the network analyzer plugin of Cytoscape 3.8.2. Hub nodes were identified based on node degree distribution. The network was clustered using the Glay (community cluster) Cytoscape plugin app from clusterMaker for network clustering (with undirected edges). GLay clusters network based on densely interacting nodes and functional relevance of nodes.

### Construction and analysis of protein–protein interaction (PPI) network

While gene interaction network is used to identify hub genes, analysis of protein–protein interaction network allows us to assess the corresponding protein interactions inside the cells at a molecular level. PPI network was constructed using an online database named Search Tools for the Retrieval of Interacting Protein (STRING; version 11.0; https://string-db.org) which furnishes validated as well as predicted functional protein association network data. We imported the list of protein encoding genes from co-expression network clusters in the STRING database to observe the functional interaction relationship among them, with the cut-off interaction score for the network set to > 0.400 (medium confidence). To visualize the PPI network, Cytoscape software (version 3.8.2; https://cytoscape.org) was used. We identified hub proteins from the PPI networks of each cluster based on node degree (number of edges connected between protein nodes), which are the most highly connected nodes with key biological functions. GeneCodis4, an online web server was used to perform functional analysis for the GO and KEGG pathway of the proteins in the PPI network.

### Analysis of LncRNA-transcription factor-gene regulatory network

To construct a TF-Gene interaction regulatory network, we retrieved data from Open-Access Repository of Transcriptional Interaction database (ORTI, http://orti.sydney.edu.au/index.html) which consists of experimentally validated transcriptional interactions. ORTI database consists of HTRI (chromatin immunoprecipitation followed by deep sequencing data) database which harbors TF-TG (driver genes) interactions data. Another dataset of lncRNAs-TFs and lncRNAs- driver genes regulatory interactions has been generated through manual text mining with due focus on quality dataset. All ambiguous data were discarded. This dataset harbors a collection of lncRNA-target regulatory relationships validated from low-throughput and high-throughput experimental methods and these regulatory interactions were included in the network. All these data were imported in Cytoscape 3.8.2 and merged into one master network. Quantitative directed network analyses were done on this lncRNA-TF-mRNA (driver genes) regulatory interaction network using lncRNA/TFs as a source to target driver genes. We further predicted hub bottleneck lncRNA-coding gene interaction sites using a validated human lncRNA-mRNA interaction database (http://rtools.cbrc.jp/cgi-bin/RNARNA/index.pl) which contains a large data of lncRNA-mRNA and lncRNA-lncRNA interaction. This database provides the location of the lncRNA interaction sites on coding genes along with their binding energy.

### Significance

LncRNA and mRNA pan-cancer regulatory network modeling identifies EGR1-*MALAT1*-coding genes regulatory axis with possible implication in multi-drug resistance. This approach may be used to understand how lncRNAs-mRNA regulatory interactions can influence pan-cancer multi-drug resistance, and improve targeted therapy.

## Results

### Pan-cancer identification of drug-sensitive and -resistant NRAS-mutant cell lines for select drugs

To identify individual drug-resistant and sensitive cell lines for each drug, we studied dose–response curves of all available drugs taken from GDSC. It was observed that 41 *NRAS*-mutant cancer cell lines were commonly responsive to 10 drugs, namely PD-0325901, Trametinib, Selumetinib, TL-1-85, CI-1040, NG-25, PLX4720, AP-24534 (Ponatinib), Xl-184 (Cabozantinib) and Foretinib. We collected the normalized IC_50_ value of these 41 cancer cell lines from the GDSC database for the 10 drugs and performed uncentered hierarchical clustering and generated the heatmap to cluster the drug-sensitive and -resistant cell lines. All the cell lines were derived from different cancer types classified at the TCGA matching label (Supplementary Table S2a).

**Figure 1:**
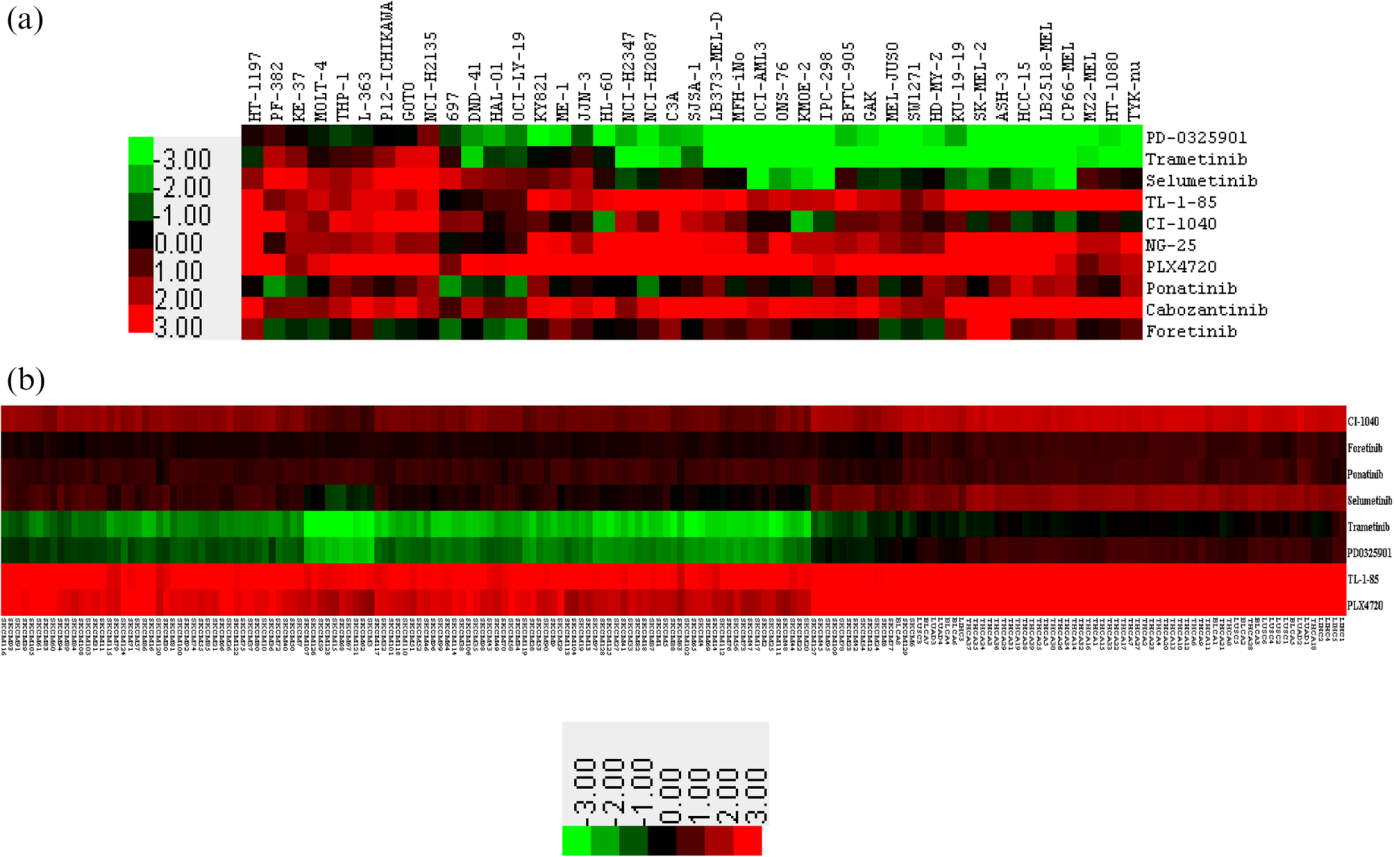
(**a**) Heatmap of drug dose–response in cell lines. Patterns of log normalized drug IC_50_ value clustered together. Rows represent drugs and columns represent cell lines. Drug-sensitive cell lines are shown in green and drug-resistant cell lines are shown in red color. (**b**) Heatmap of drug response in cancer tissues. Predicted drug IC_50_ value clustered together. Rows represent drugs and columns represent cancer tissue samples from patients. Drug-sensitive cancer tissue samples are shown in green and drug-resistant cancer tissue samples are shown in red color.

The heatmap colors represent dose–response in terms of the IC_50_ value of a particular drug across cell lines. Each column in the heatmap represents cell lines and each row indicates the normalized IC_50_ score for a compound. Cell lines with normalized IC_50_ value greater than 0 were considered as drug-resistant cell lines and cell lines with normalized IC_50_ value less than 0 were referred to as sensitive cell lines in response to the drug^38^ (Fig. 1a). We selected the drug-sensitive (IC_50_ < -1) and -resistant (IC_50_ > 1) *NRAS*-mutant cancer cell lines based on IC_50_ value and color intensity and enlisted these **(**Supplementary Table S2b). As shown in the heatmap, there were no individual drug-sensitive cell lines found for four drugs (TL-1-85, NG-25, Cabozantinib, and PLX4720), on the other hand, in the case of PD-0325901, we found only one drug-resistant cell line.

### Correlation and validation of drug IC50 data from cell lines with cancer tissues

We wanted to further investigate if there existed any similarities in the pattern of drug-resistance and sensitivity in cancer cell lines and cancer tissue samples. A similar correlation pattern would indicate a similarity in gene expression pattern that may be responsible for causing drug-resistance or drug-sensitivity. We compared the drug-response heatmaps **(**Fig. 1a,b) generated for cancer cell lines and molecular data generated from cancer tissue samples from patients. Most of the cancer tissue samples exhibited resistance and sensitivity for all the drugs except in the case of cancer tissue samples from SKCM, where the tissue samples exhibited Trametinib- and PD-0325901–specific drug sensitivity while few SKCM tissue samples also exhibited drug sensitivity towards Selumetinib. *NRAS*-mutant cancer tissue samples were not available in TCGA for LAML, ALL, MM, MB, NB, DLBC and SCLC cancer types.

### FN1, CD44 and TIMP3 are among the nine genes most significantly differentially expressed between drug-sensitive and -resistant cancer cell lines and common across multiple drugs

**Figure 2:**
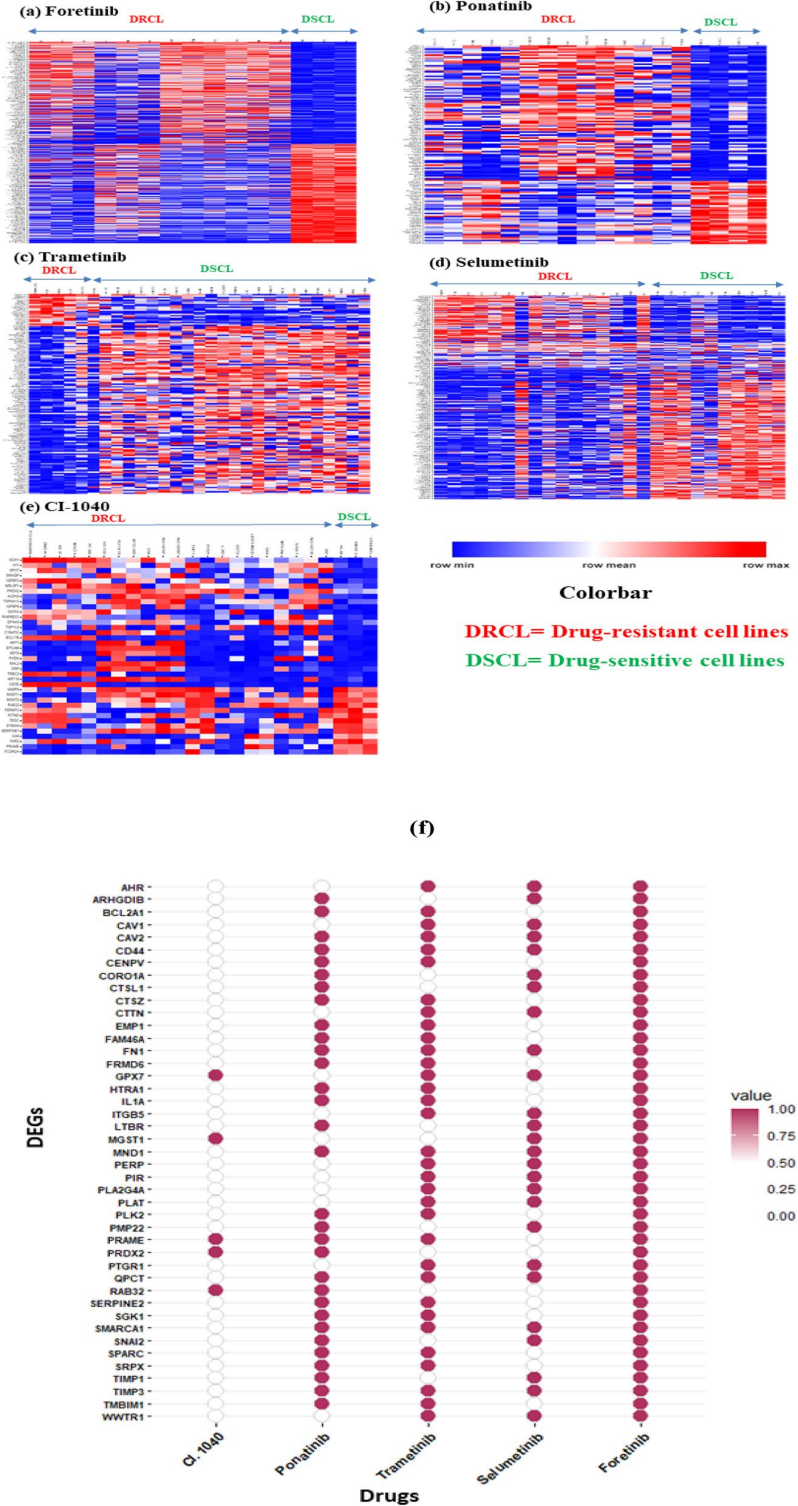
Differential gene expression analysis between drug-sensitive and -resistant cancer cell lines. (**a**–**e**) Heatmap of DEGs between drug-sensitive and -resistant cancer cell lines. Expression pattern of DEGs in cancer cell lines for each drug is shown. Red color: up-regulated genes, blue color: down-regulated genes. (**f**) Genes that are differentially expressed (reddish-brown bubble) between drug-sensitive and -resistant cancer cells in the case of multiple drugs.

Several studies have revealed that gene expression profiling reveals several important predictive biomarkers in drug-sensitivity and resistance studies^12,13,39^. To identify and analyze significant DEGs that may be involved in drug sensitivity/resistance from our dataset, we used the normalized basal gene expression profiling data from the GDSC database associated with pan-cancer drug sensitivity and resistance. Several DEGs were identified that are differentially expressed between drug-sensitive and -resistant cancer cell lines (for pan-cancer *NRAS*-mutant cell lines) using Welch’s t-test (unequal group variance) with threshold *p* < 0.05, for 5 drugs. For the other 5 drugs, we did not perform this statistical test because as seen from Supplementary Table S2b, all of the chosen cancer cell lines were either uniformly sensitive (PD-0325901) or uniformly resistant (TL-1-85, NG-25, Cabozantinib, PLX4720) to these drugs and so, no differential effect can be seen in the individual cases. To statistically validate further, we applied double filtration (*p* value < 0.05, log2FC > 2) and generated volcano plots in the case of each drug (Supplementary Fig. S3b). The number of significantly differentially expressed genes was found to vary from 38 (CI-1040) to 467 (Foretinib) across 5 drugs (Supplementary Table S2c). As the volcano plot shows, significantly differentially expressed genes are shown as top blue dots and up-regulated and down-regulated genes in resistant cancer cell lines at right and left position, respectively (*p* < 0.05, log2 FC > 2). To validate further, we used an uncentered hierarchically-clustered drug *vs* cell lines heatmap generated above to discriminate differentially-expressed genes from these cell lines using comparative marker selection in GenePattern (version 10.1) **(**Fig. 2a–e). It was observed that the results more or less coincided with our volcano plots.

We next scouted for the common differentially expressed genes between drug-sensitive and -resistant cancer cell lines across these 5 drugs. A total of nine genes including *FN1, TIMP3,* and *CD44* were observed to be common across four drugs. Similarly, genes including *SPARC, TIMP1,* and *SNAI2* were observed to overlap across three drugs (Fig. 2f). These predictive analyses of gene expression data from the GDSC database revealed key significantly differentially expressed genes (DEGs) strongly associated with drug sensitivity or resistance, and we proceeded further using our narrowed down gene list to identify the biological processes involved as well as to perform integrated network analyses to identify key hub genes and their regulation.

### Functional enrichment analysis through Gene Ontology (GO) and KEGG Pathway inform signal transduction, proteoglycans (pathway) in cancer as one of the major pathways involved

Given this set of DEGs across multiple drugs naturally, the next step is to discern functional enrichment of biological processes and KEGG pathways. We used the web tool, GeneCodis4, to annotate likely biological processes and the KEGG pathway, with a default cut-off *p*-value (FDR) < 0.05. Gene ontology terms for biological processes DEGs significantly enriched for the drug Ponatinib include GO: 0007165- signal transduction, GO: 0006915-apoptotic process, and GO: 0006508 proteolysis, GO: 0008285-cell cycle, GO: 0006355-regulation of transcription, DNA-dependent and cell division (Supplementary Fig. S4).

In addition, KEGG pathway analyses revealed that DEGs were significantly enriched in hsa04151:PI3K-Akt signaling pathway, hsa05200: Pathway in cancer, has04510: Focal adhesion, and hsa01100: Metabolic pathway (Supplementary Fig. S4).

In the case of the other four drugs, the DEGs were enriched in biological processes and KEGG pathways were more or less the same. GO terms include GO: 0007165 signal transduction, GO: 0007155 cell adhesion, GO: 0006915 apoptotic processes, GO: 0006508 proteolysis and KEGG pathways include hsa05205 proteoglycans (pathway) in cancer, hsa05200: Pathway in cancer, has04510: Focal adhesion, and hsa01100: Metabolic pathway (Supplementary Fig. S4).

### Gene co-expression network analysis of DEGs identifies FN1 among the top hub genes

**Figure 3:**
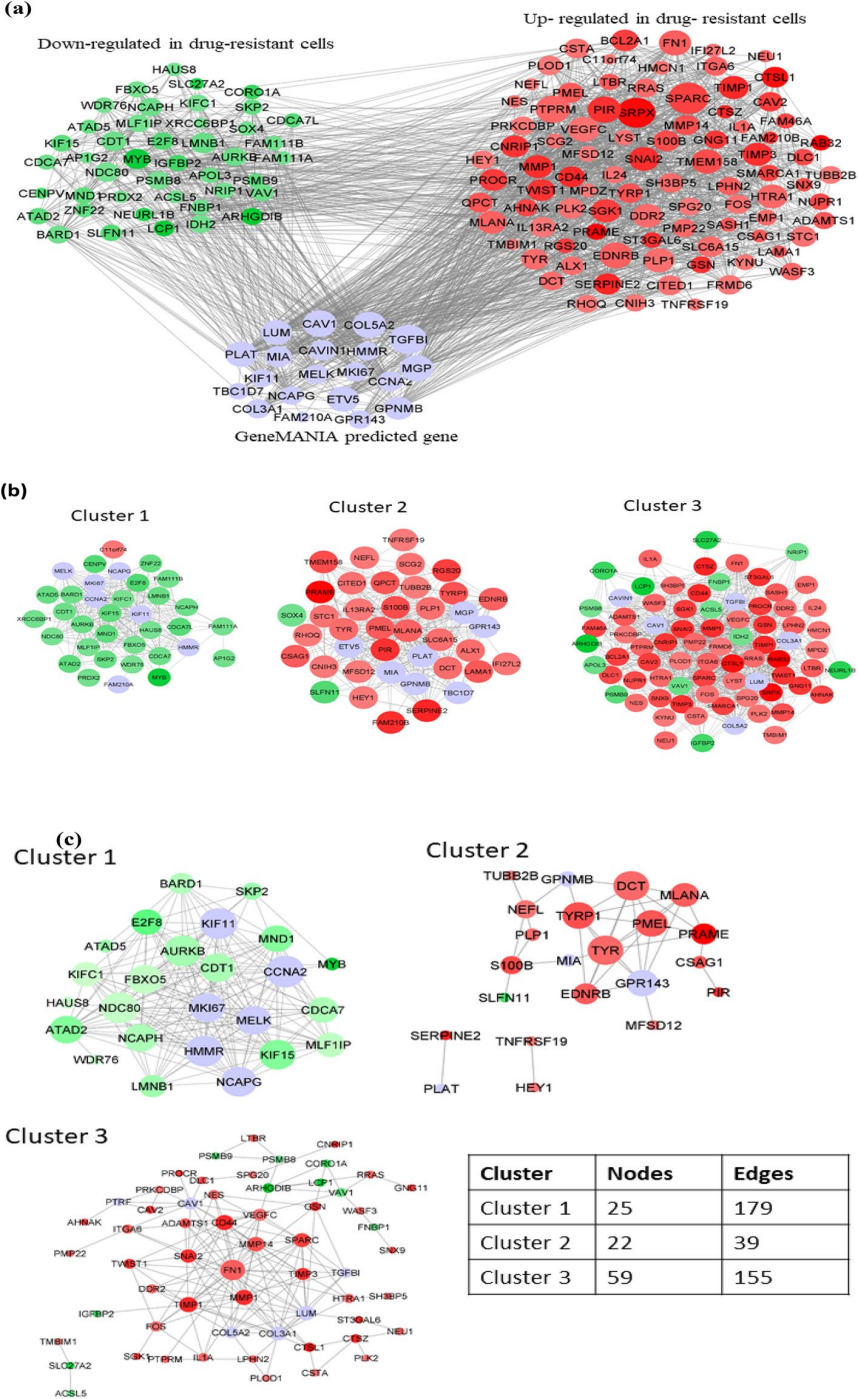

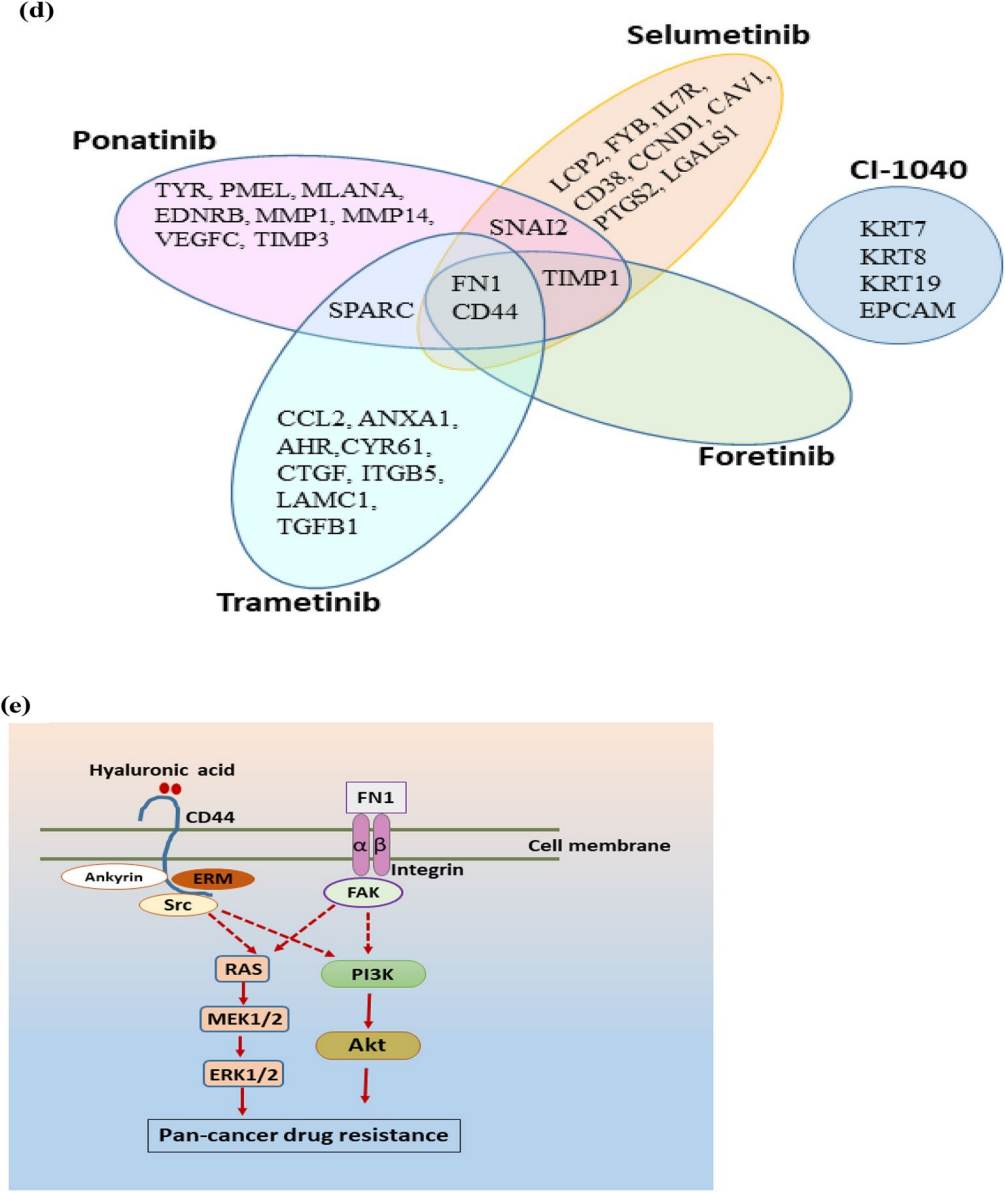
Gene co-expression and PPI network. (**a**,**b**) Gene co-expression network of DEGs for Ponatinib predicted from GeneMANIA and visualized in Cytoscape. The co-expression network represents up- (red) and down-regulated genes (green) and GeneMANIA-predicted genes (blue); the top hub genes were detected by node degree (node size proportional to node degree) is seen in (**a**); (**b**) depicts Glay generated modules: cluster 1, cluster 2 and cluster 3. C-D: PPI network of clusters. (**c**) Detection of hub proteins by analyzing node degree for Ponatinib (red nodes: up-regulated; green nodes: down-regulated, blue nodes-GeneMANIA predicted). Circle size is proportional to the node degree. (**d**) Venn diagram of common hub protein-coding genes identified across the drugs. (**e**) Diagrammatic representation of the interconnectivity of topmost genes FN1 and CD44 in RAS and PI3K/Akt signaling pathways taken from KEGG. Dashed arrows represent several other proteins involved in signaling. Data are taken from KEGG Pathways (CD44 in signaling: https://www.kegg.jp/kegg-bin/highlight_pathway?scale=1.0&map=map05205&keyword=cd44; FN1 in signalling: https://www.kegg.jp/kegg-bin/highlight_pathway?scale=1.0&map=map04151&keyword=fn1).

In order to undertake deeper functional analysis and to assess possible molecular interactions of these identified DEGs in drug-resistant cancer cell lines at the gene level, we constructed and analyzed the co-expression network of DEGs and GeneMANIA-predicted genes for the five drugs enlisted from our previous analysis above. The generated networks are shown in Fig. 3a and quantitative analyses in Supplementary Table S3. The number of nodes and edges for the respective networks are shown in Table 1. From the quantitative network analyses, in the case of Ponatinib, top 34 hub genes were identified based on the highest node degree from the co-expression network (Supplementary Table S3). This network was then clustered into three modules using Glay (community cluster), a Cytoscape plugin. The identified clusters 1, 2, and 3 contain 35 nodes, 40 nodes, and 77 nodes, respectively (Fig. 3b). Similarly for other drugs; 52 hub nodes for Foretinib, 48 hub nodes for Selumetinib, 35 hub nodes for Trametinib, and 13 hub nodes for CI-1040 (Table 1, Supplementary Table S3) were identified. Then, the network clustering generated different clusters in case of other four drugs: 6 clusters for Foretinib, 4 clusters for each Selumetinib and Trametinib, 3 clusters for CI-1040 (Supplementary Fig. S5). After our gene networks generation, we proceeded to generate protein–protein interaction networks in order to assess whether the same hub genes can also be found as hub proteins.

**Table 1:** List of number of nodes/edges and identified hub nodes in gene co-expression network for each of the five drugs.

Drug name	Number of nodes in network	Number of edges in network	Number of hub nodes	Lowest node degree for hub node selection
Ponatinib	152	1826	34	30
Foretinib	483	14,156	52	100
Selumetinib	256	2410	48	30
Trametinib	165	3019	35	50
CI-1040	49	211	13	10

### Protein–protein interaction network analysis of DEG-encoded proteins shows FN1 and CD44 among the key hub proteins

Identification of key driver/hub proteins in the DEGs-encoded protein–protein interaction (PPI) network in drug-resistant cancer cell lines provides an important source of interpretation of the regulatory mechanisms in drug-resistant cancer. We defined driver gene/protein as the one which is prominently associated with drug resistance and acting as a hub gene/protein. To study the same interactions at the functional protein level, and assess whether the gene and protein networks coincide in their overall pattern, PPI network of genes from each clusters of the gene co-expression network for all the five drugs using STRING were constructed at median confidence score of 0.400, which quantifies the strength of corroborative evidence for the reported interactions in the PPI network^40^. The analyzed network for Ponatinib is shown in Fig. 3c and for other drugs, respective networks are shown in Supplementary Fig. S6a. Top hub proteins from all PPI networks generated were identified and are mentioned in the Supplementary Tables S4 and S5. We have also performed functional analysis of these proteins enriched in various GO terms and KEGG pathways (Supplementary Fig. S6b). The PPI networks were observed to comprise 10 hub proteins for four drugs (Supplementary Table S5), whereas for drug CI-1040, only four hub protein nodes were identified. We were not able to acquire PPI networks for some of the clusters because of the lack of data for these clusters in STRING.

The node proteins from the PPI networks of the drugs studied were significantly enriched in distinct GO terms and KEGG pathways. These significant functional pathways belonged to cell migration, cell population proliferation, signal transduction, melanogenesis, cell adhesion, cytokine-mediated signaling and proteolysis for biological processes and from KEGG, we observed proteoglycans pathway and PI3K-Akt signaling pathways in cancer.

Upon further analysis, combining PPI networks and gene co-expression networks, common hub protein-coding genes were selected. For Ponatinib, there were nine and four hub proteins for cluster3 and cluster2 respectively, with shared common genes among the hub gene list from gene co-expression network and the STRING-generated top hub proteins (Table 2). Through chronological analyses, we were able to identify TYR, PMEL, MLANA, EDNRB, FN1, CD44, MMP1, TIMP1, MMP14, TIMP3, SPARC, SNAI2, and VEGFC as key hub proteins for Ponatinib common between co-expression and PPI network hub node. Similarly for other drugs, FN1, TIMP1 and CD44 for Foretinib, LCP2, FYB, IL7R, CD38, FN1, CD44, TIMP1, CCND1, CAV1, PTGS2, SNAI2 and LGALS1 for Selumetinib, CD44, CCL2, ANXA1, AHR, FN1, SPARC, CYR61, CTGF, ITGB5, LAMC1 and TGFB1 for Trametinib, KRT7, KRT8, KRT19 and EPCAM for the drug CI-1040, were identified as common hub proteins between co-expression and PPI network hub node list.

**Table 2:** List of common hub nodes between gene co-expression and PPI network for each five drugs.

Drug name	Cluster	Common hub nodes between gene co-expression and cluster PPI network
Ponatinib	Cluster 1	-NA-
	Cluster 2	TYR, PMEL, MLANA, EDNRB
	Cluster 3	**FN1, CD44**, MMP1, **TIMP1**, MMP14, **SPARC**, **SNAI2**, VEGFC, TIMP3
Foretinib	Cluster 2	**FN1, TIMP1, CD44**
Selumetinib	Cluster 2	LCP2, FYB, IL7R, CD38
	Cluster 3	**FN1**, **CD44**, **TIMP1**, CCND1, CAV1, PTGS2, **SNAI2**, LGALS1
Trametinib	Cluster 1	**CD44**, CCL2, ANXA1, AHR
	Cluster 2	**FN1**, **SPARC**, CYR61, CTGF, ITGB5, LAMC1, TGFB1
CI-1040	Cluster 2	KRT7, KRT8, KRT19, EPCAM

We next wanted to find common hub proteins across all the drugs studied. Among the hub protein nodes from PPI and co-expression networks as mentioned above, FN1 and CD44 were found to be common for 4 drugs; Ponatinib, Foretinib, Selumetinib and Trametinib. TIMP1 was common in the case of three drugs (Selumitinib, Ponatinib, Foretinib) while SPARC (Ponatinib and Trametinib) and SNAI2 (Ponatinib and Selumetinib) were common for two drugs, respectively (Fig. 3d). These hub proteins might be inducing drug-resistance in cancer through many divergent pathways, including proteoglycans pathway in cancer, focal adhesion pathway, metabolic and PI3K-Akt signaling pathways. Therefore, targeting these hub proteins is one likely mechanism to inhibit the upstream and downstream pathways in biological processes to curb the pan-cancer drug resistance. The interconnectivity of key hub proteins FN1 and CD44 with RAS and PI3K/Akt signalling pathways, taken from KEGG Pathways, is shown in Fig. 3e.

### Gene-regulatory modules: MALAT1 and EGR1 form an interaction loop to regulate key hub genes

**Figure 4:**
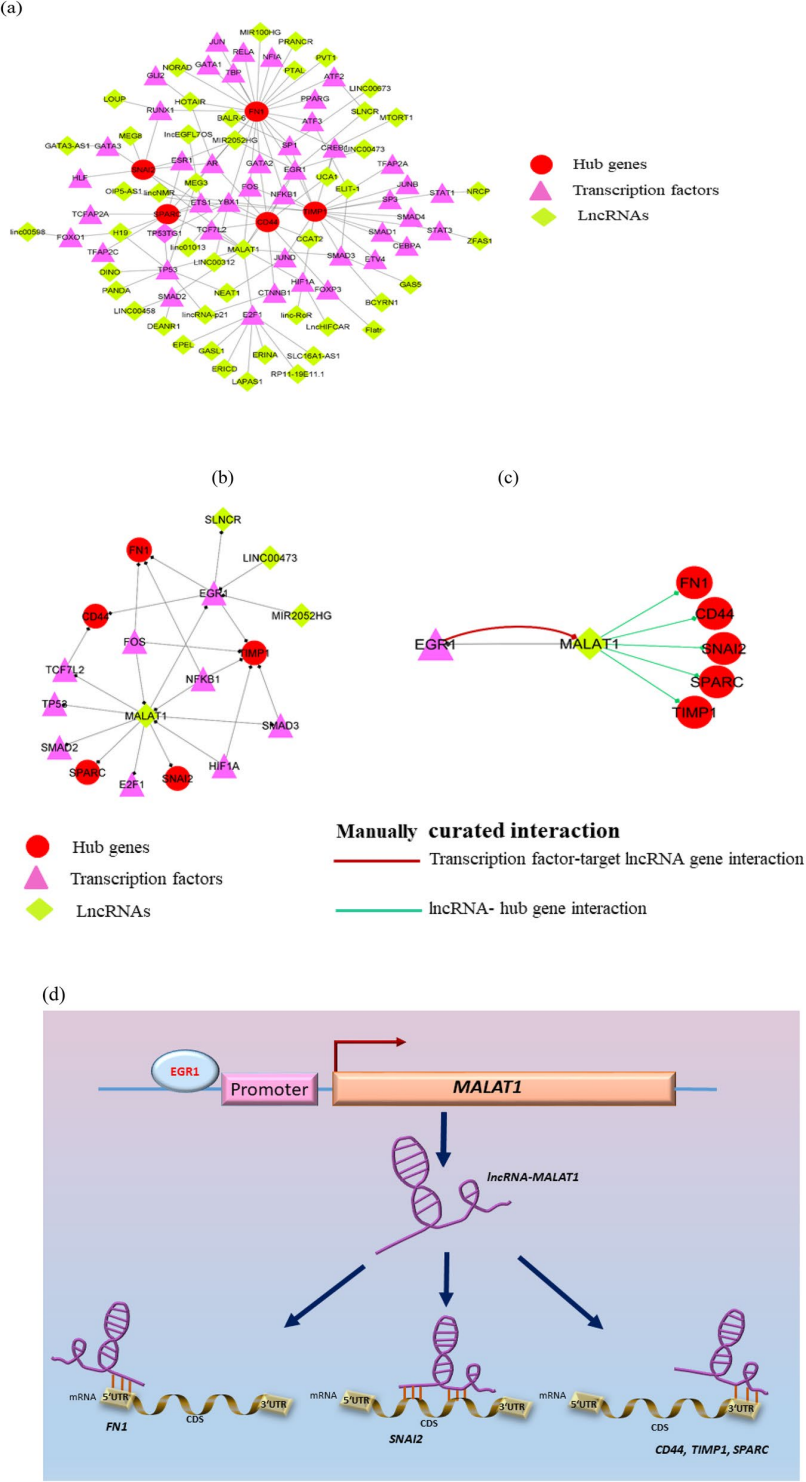
Regulatory network of LncRNAs-TFs-Driver genes and working model (**a**) Master regulatory network of LncRNA-TF-Driver genes. Regulatory interactions between lncRNAs, TFs and driver genes are depicted in this integrated network, b-c:EGR1 and *MALAT1 s*ubnetwork from the master regulatory network. (**b**) Transcription Factor EGR1 is observed to be regulating three hub genes (*FN1, CD44* and *TIMP1)* and also being regulated by *MALAT1* (**c**) EGR1-*MALAT1* interaction (red-colored edge) and *MALAT1*-mRNA interaction (hub genes, green-colored edges) obtained from the ENCODE transcription factor targets dataset of the Harmonizome database and lncRNA-mRNA interaction database, respectively, (**d**): A working model for the regulation of key driver genes associated with pan-cancer multi-drug resistance: Transcription factor EGR1 may regulate *MALAT1* transcription*.* After being transcribed, *MALAT1* is predicted to bind to different sites, 5′-UTR, CDS, and 3′-UTR, of specific mRNAs, thereby regulating these key driver genes through *cis-*or *trans-*acting mechanisms.

While identifying druggable genes/proteins involved in pan-cancer drug resistance is an important step, it is also critical to fully understand the regulatory mechanisms occurring to regulate these genes, if we are to target them. LncRNAs, apart from proteins, are another arm of the emerging regulatory mechanisms in drug resistance. To interrogate the specific lncRNAs regulating our enlisted hub genes/proteins, a lncRNA-TF-mRNA (hub gene) interaction network was constructed and analyzed using Cytoscape. The relevant data for TF and regulated hub genes were retrieved from the ORTI database. We also performed literature search for other lncRNAs interacting with TFs and hub genes. A comprehensive regulatory interaction gene network was generated that consists of a total of 91 nodes (genes/proteins) and 125 edges (interaction between the nodes) including 48 lncRNAs, 38 TFs, and 5 hub protein-coding genes (Fig. 4a). We analyzed the network based on two parameters, node degree (outdegree), and betweenness centrality (node characterized by having shortest path passing through it). In the directed network analysis, it was observed that among lncRNAs, *MALAT1* had the highest node degree and betweenness centrality. Among TFs, EGR1, AR and YBX1 had the highest node degree, and EGR1 had the highest betweenness centrality among these three TFs. The other lncRNAs with the highest out-degree and betweenness centrality were *HOTAIR* and *lincRNA-p21,* respectively Table 3. From our network analysis, we observed that the transcription factors EGR1, AR, and YBX1 regulate *CD44,* and *FN1*; YBX1 and AR regulate *SPARC;* and EGR1 and YBX1 regulate *TIMP1*. Further, we observed that lncRNA *MALAT1* might regulate directly, two hub genes (*SPARC* and *SNAI2*) and six transcription factors including EGR1, as depicted from the regulatory subnetwork. It was also observed that *MALAT1* indirectly regulated three hub genes (*FN1, CD44,* and *TIMP1*) via the transcription factor EGR1 (Fig. 4b). The regulation between the lncRNA *MALAT1* and the transcription factor EGR1 is a kind of mutual interaction as can be depicted from the ENCODE transcription factor target dataset taken from the Harmonizome database (http://amp.pharm.mssm.edu/Harmonizome).

**Table 3:** Quantitative analyses of LncRNA-TFs-Gene (hub gene) regulatory network based on out-degree and betweenness centrality.

Sr no.	Gene name	Outdegree
1	*MALAT1*	8
2	*EGR1*	4
3	*AR*	4
4	*YBX1*	4
5	*NFKB1*	3
6	*FOS*	3
7	*ETS1*	3
8	*HOTAIR*	3
9	*H19*	3
10	*HIF1A*	2

Sr no.	Gene name	BetweennessCentrality
1	*MALAT1*	0.336730123
2	*HIF1A*	0.274132139
3	*TP53*	0.205823068
4	*EGR1*	0.174412094
5	*YBX1*	0.168868981
6	*lincRNA-p21*	0.138969765
7	*ETS1*	0.063493841
8	*CTNNB1*	0.051175812
9	*SLNCR*	0.044456887
10	*SP1*	0.039193729

### Mode of MALAT1 action on key driver genes

In order to decipher the *cis* and *trans* regulatory actions of *MALAT1* on these select target genes, we collected the genomic location information of these coding hub genes as well as *MALAT1* from NCBI Gene (https://www.ncbi.nlm.nih.gov/gene/) database. We surmised that, due to the same location at chromosome 11 of *MALAT1* and *CD44* (Supplementary Table S6a), *MALAT1* may regulate *CD44* transcription through *cis*-acting mode of action. Chromosome 11 is among the most gene- and disease markers-rich chromosomes in humans. *MALAT1* may regulate the other four hub genes (*FN1, TIMP1, SNAI2* and *SPARC)* through *trans*-acting mode of action, owing to their differing chromosomal locations.

We further wanted to check whether *MALAT1* may regulate these hub genes post-transcriptionally by directly interacting with their mRNAs. For this purpose, we obtained the predicted interactions of the lncRNA *MALAT1* with the hub gene mRNAs, from the lncRNA-mRNA interaction database (Fig. 4c). The interaction patterns suggest that *MALAT1* interacts with the hub gene mRNAs at different interaction sites. For instance, *MALAT1* is observed to interact with *CD44* and *SPARC* at the 3´UTR region. It is also observed to interact with *TIMP1,* and *SNAI2* at the CDS region, as well as with *FN1* at the 5´UTR (Supplementary Table S6b). This suggests that post-transcriptional regulation of these genes is also possible, as seen in several studies with other genes in the literature.

Hence, this EGR1-*MALAT1*-coding genes regulatory axis may regulate pan-cancer multi-drug sensitivity/resistance through either or both *cis* and *trans*-acting mechanisms at the transcriptional and post-transcriptional level.

## Discussion

Mutant *NRAS* is an intractable target indirectly and frequently involved in drug resistance in multiple cancer types. In an attempt to uncover connected signaling molecules with possible involvement in drug sensitivity or resistance, we performed a systematic big data analysis of coding genes in a pan-cancer context and their regulation by long non-coding RNAs, utilizing drug dose–response of 5 drugs for *NRAS*-mutant cancer cells lines.

Upon identification of key differentially expressed genes between drug-sensitive and -resistant cancer, the GO terms for biological processes significantly enriched from this set of DEGs for most of the drug cases were: signal transduction, cell adhesion, apoptotic process, proteolysis, and cell cycle, and KEGG pathways gene set were enriched in proteoglycans pathway in cancer, focal adhesion pathway, metabolic pathway and PI3K/Akt signaling pathway. As these pathways are found overrepresented in our studies, this functional enrichment analysis revealed that these genes likely play a major role in oncogenesis and drug resistance in cancer. Further analyses, utilizing gene co-expression and PPI network clusters, confirmed similar functional modules of biological processes and the KEGG pathway. Several studies such as Lee et al.^8^ and others have focussed on particular pathways involved in drug resistance using integrative meta-analysis.

Unifying the mRNA concentration and protein abundance profiles is of major importance in effective therapeutic biomarker identification. The construction of a PPI network in addition to gene co-expression network allows us to assess the functional role of a protein encoded by a hub gene. In parallel with the gene co-expression network analyses, hub (driver) proteins were identified from respective functional clusters of the PPI network for the drugs common with the co-expression hub gene list. The study identified *FN1, CD44, TIMP1, SPARC* and *SNAI2* as common coding hub proteins for most of the drug-resistant cancer types. Driver genes/proteins were identified based on the topmost node degree from these networks. From heatmap analyses, it was observed that all of these protein-coding hub genes were up-regulated in the case of Ponatinib-resistant cancer and only *FN1, CD44* and *TIMP1* were up-regulated in Foretinib-resistant cancer. However, in the case of combined Selumetinib- and Trametinib-resistant cancers, *FN1* and *CD44* were down-regulated; *TIMP1* and *SNAI2* were down-regulated only in Selumetinib-resistant ones; while *SPARC* was down-regulated in the case of Trametinib-resistant cancer. Previous studies have suggested that some of these identified hub genes function as biomarkers in a variety of cancer types such as breast, head and neck and serous ovarian cancer^41,42^. Previous studies have observed significant *FN1* upregulation in tumors resistant to doxorubicin^43^. *FN1* is also found to play an important role in the activation of the Akt signaling pathway in drug-resistant cancer^44^. On the other hand, *CD44* is known to be a proteoglycan that plays an important role in cell–cell and cell–matrix adhesion by binding to fibronectin^45^. Cancer cells with acquired drug-resistance possess a higher expression of CD44s isoform, which may play a role through regulation of multiple signaling pathways^46^. TIMPs, which include *TIMP1*, are secreted proteins that play a crucial role in cancer progression and invasion^47,48^. *TIMP-1*, overexpressed across almost all cancer types, is found to protect cells against chemotherapy-induced apoptosis^49^. *SNAI2* is a member of the Snail family of zinc finger transcription factors, and is observed to be highly expressed in Fulvestrant-resistant and Tamoxifen-resistant breast cancer^50^. It is found to have an implication in several human malignancies^51^. Similarly, *SPARC* is known to act as an oncogene in certain cancer types and is reported to act as a tumor suppressor in other cancer types^52^.

Our studies then focused on identifying master regulators of these hub genes involved in drug resistance. LncRNAs have been associated with drug sensitivity and resistance in cancers and have been found to act as prognostic molecules which can modify chemosensitivity^53–55^. Therefore, we wanted to identify key lncRNAs which could regulate our identified key driver genes from co-expression and PPI network studies, in order to alter their expression in drug-resistant cancer.

From the directed network analyses of lncRNA-TFs-mRNA (driver genes) interaction regulatory network, we have identified lncRNA *MALAT1* to be the major interacting component based on network parameters. *MALAT1* may bind to the corresponding mRNAs of these driver genes at 5′ UTR of *FN1*; CDS of *TIMP1, SNAI2*; and 3′ UTR of *CD44* and *SPARC*, and also may regulate their expression through the proven mechanisms of actions such as mRNA splicing^56^. These mechanisms are among the major ones functional in drug-resistant cancers^57,58^. Moreover, *MALAT1* is a widely studied lncRNA originally reported to be associated with metastasis in the early stage of non-small cell lung cancer^57,59^ and is subsequently found to be involved in a variety of cancers. *MALAT1* is transcribed from human chromosome 11q13.1 and the transcript is localized in the nuclear speckles, a site for pre-mRNA splicing^60^. Driver genes *SPARC* and *SNAI2* that were observed to be interacting with *MALAT1* from our network interaction study*,* were found to be down regulated in *MALAT1*-depleted breast cancer samples^61^. Results from our studies using harmonizome ChIP-Seq data show that transcription factor EGR1 could transcriptionally regulate *MALAT1*. We predict that EGR1 and *MALAT1* might be regulating each other through a feedback loop regulatory system. Further, as *MALAT1* and one of the select protein-coding genes, *CD44,* are located on the same chromosome 11, *MALAT1* might be regulating *CD44* in a *cis*-regulatory manner. Other select coding genes, *FN1, TIMP1, SNAI2, SPARC*, reside at a different chromosomal location than *MALAT1*, and, therefore, could be regulated in a *trans*-regulatory manner. This further confirms the prevailing widespread notions of two plausible scenarios, first, that many lncRNAs can interact with or regulate one coding gene at a time, and second, that many coding genes may be regulated by one or multiple lncRNAs simultaneously.

Converging our above studies, we propose a working model of the mechanism of regulation of the select driver genes in pan-cancer (*NRAS*-mutant cancers) drug-resistance. This model features a regulatory axis comprising EGR1-*MALAT1*-driver genes (*FN1, CD44, TIMP1, SPARC,* and *SNAI2*) (Fig. 4d). Specifically, transcription factor EGR1 may regulate *MALAT1* transcription, since both these genes are observed to express at a similar level in Cisplatin-resistant cancer^62^. After being transcribed, *MALAT1* is predicted to bind to different sites at 5′-UTR, CDS, and 3′-UTR, of specific mRNAs, thereby regulating these key driver genes through either *cis-* or *trans-*acting mechanisms.

Taken together, our analyses suggest that these driver genes may be overexpressed or repressed via direct or indirect interactions with *MALAT1* leading to drug resistance/sensitivity in context. Literature studies also corroborate our findings. Of note, from our comprehensive studies, we nominate few coding and non-coding genes as key targets that can overcome or replace recalcitrant *NRAS* as a drug target.

Our findings provide key insights that may help predict drug treatment response and enhance our understanding of pan-cancer multi-drug resistance in the context of mutant *NRAS* signaling and any other selected signaling pathways. Further studies are required to be done to assess the clinical relevance of identified drug targets as therapeutic targets in *NRAS*-mutant cancer types, as these data have been taken from cell lines and some tissue samples, and because tumor microenvironment is also found to play a role, so, these may vary in vivo. Top-ranked key driver genes and lncRNAs can be further assessed through knock-down or induced expression in specific drug-resistant cancer cell lines. Studies on transcriptional dysregulation of lncRNA itself and the impact on regulatory activity in drug-resistant cancers are also the need of the hour.

## Conclusions

In our study, using the well validated GDSC (cell lines) and TCGA database (patient samples) which hosts molecular data from cancer patients, we performed large-scale gene expression, gene co-expression and protein–protein interaction network analyses on selected drugs inducing drug sensitivity/resistance. From our big data pan-cancer studies, among the identified hub genes (protein-coding), *FN1, CD44, TIMP1, SNAI2,* and *SPARC* were found to be common hub nodes between co-expression and PPI networks across multiple drugs, and these genes were significantly enriched in signal transduction, and PI3K/Akt-signaling pathway, among others*.* Further studies of the regulation of these hub/driver genes by lncRNAs revealed that *MALAT1* may be a key regulator of these coding genes in drug resistance, acting through transcription factor EGR1. *MALAT1* could be the pan-cancer master biomarker regulating these driver genes’ expressions at the transcriptional as well as at post-transcriptional level. These comprehensive studies provide key insights towards improving multi-drug sensitivity in a pan-cancer context. These can be deliberated further to develop more effective interventions utilizing the *NRAS* pathway for pan-cancer precision medicine therapy and to decrease the clinical burden.

## Supplementary Information

Supplementary Figure S1.

Supplementary Figure S2.

Supplementary Figure S3.

Supplementary Figure S4.

Supplementary Figure S5.

Supplementary Figure S6.

Supplementary Table S1.

Supplementary Table S2.

Supplementary Table S3.

Supplementary Table S4.

Supplementary Table S5.

Supplementary Table S6.

## Data Availability

*Datasets*: All the datasets used in this study are publicly available in GDSC, TCGA and other databases. All the procedures were performed in accordance with the relevant guidelines and regulations.
