# Supplementary material for: MALAT1 as master regulator of biomarkers predictive of pan-cancer multi-drug resistance in the context of recalcitrant NRAS signaling pathway identified using systems-oriented approach
Source: Sci Rep. 2022 May 9;12:7540. doi: 10.1038/s41598-022-11214-8 (PMC9085754; doi:10.1038/s41598-022-11214-8)
Supplement: Supplementary file 1 — Supplementary Figure S1. [file 41598_2022_11214_MOESM1_ESM.pdf]

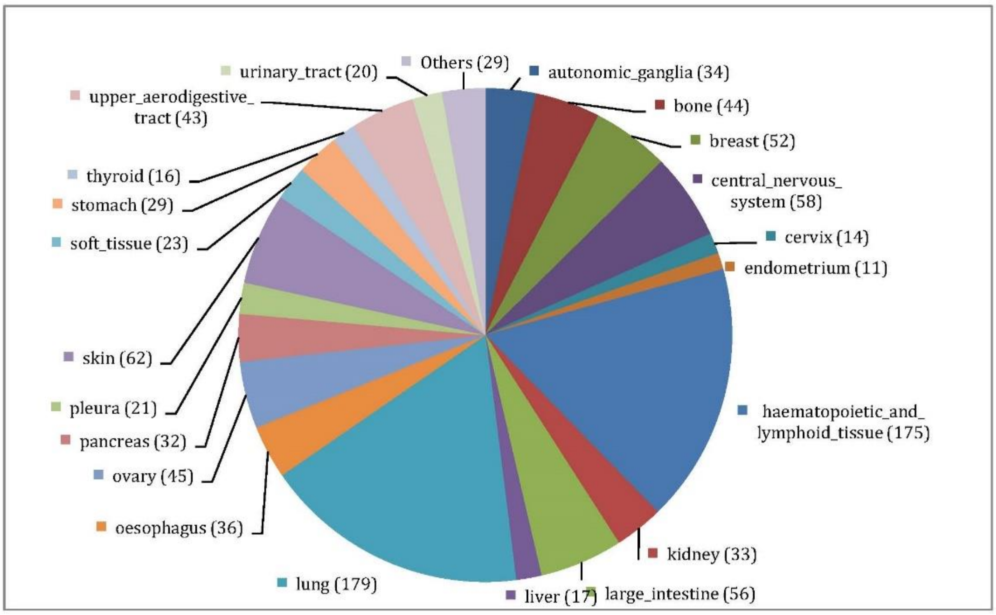

Figure S1a: Cancer cell lines derived from tissue types.

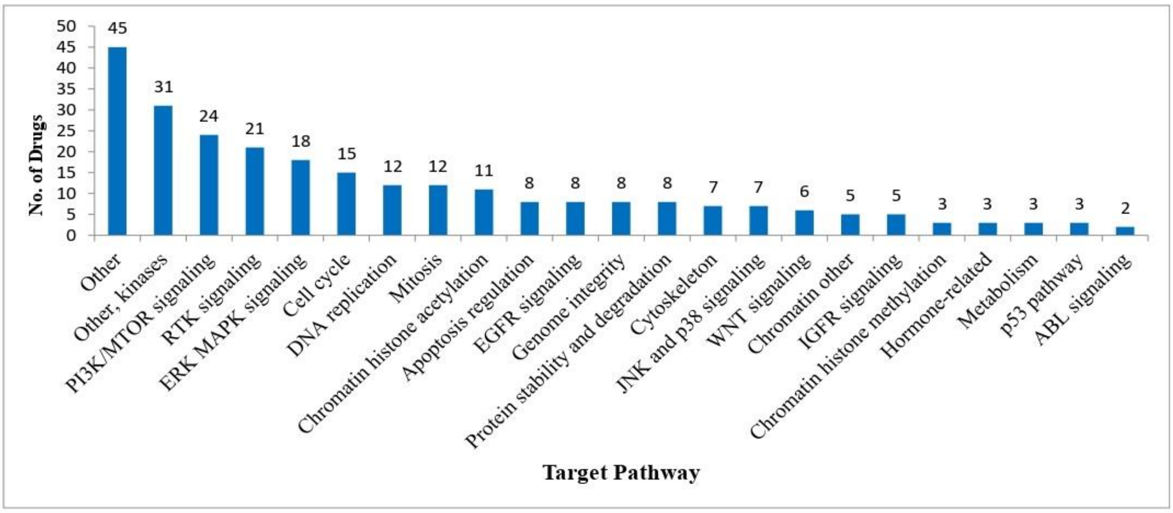

Figure S1b: **Drugs screened against cell lines targeting biological pathways.** Anticancer drugs (265 drugs) are used in screening categories based on their therapeutic targets, role in biological pathways and cellular functions. A single drug may target multiple molecules.
