# Supplementary material for: MALAT1 as master regulator of biomarkers predictive of pan-cancer multi-drug resistance in the context of recalcitrant NRAS signaling pathway identified using systems-oriented approach
Source: Sci Rep. 2022 May 9;12:7540. doi: 10.1038/s41598-022-11214-8 (PMC9085754; doi:10.1038/s41598-022-11214-8)
Supplement: Supplementary file 2 — Supplementary Figure S2. [file 41598_2022_11214_MOESM2_ESM.pdf]

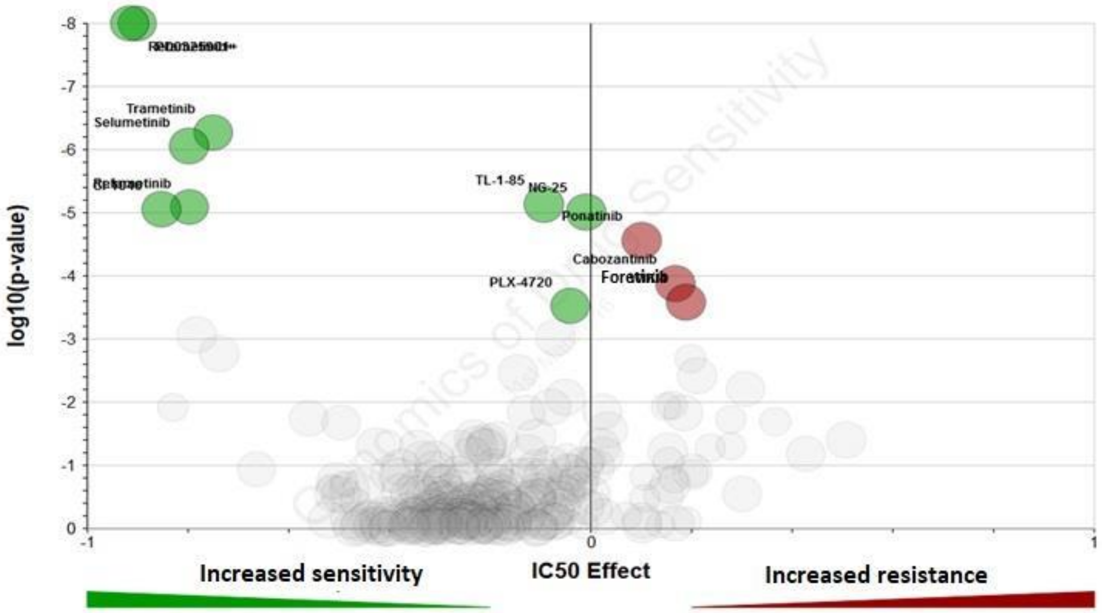

Figure S2: - Volcano plot of ANOVA analysis result taken from GDSC database. Each circle in volcano plot representing gene-drug interaction, where green circle indicating drug sensitivity & red circle indicating drug resistance. Position of circle showing how significance interaction, circle size is proportional to number of cell lines altered. (https://www.cancerrxgene.org)
