# Supplementary material for: MALAT1 as master regulator of biomarkers predictive of pan-cancer multi-drug resistance in the context of recalcitrant NRAS signaling pathway identified using systems-oriented approach
Source: Sci Rep. 2022 May 9;12:7540. doi: 10.1038/s41598-022-11214-8 (PMC9085754; doi:10.1038/s41598-022-11214-8)
Supplement: Supplementary file 3 — Supplementary Figure S3. [file 41598_2022_11214_MOESM3_ESM.pdf]

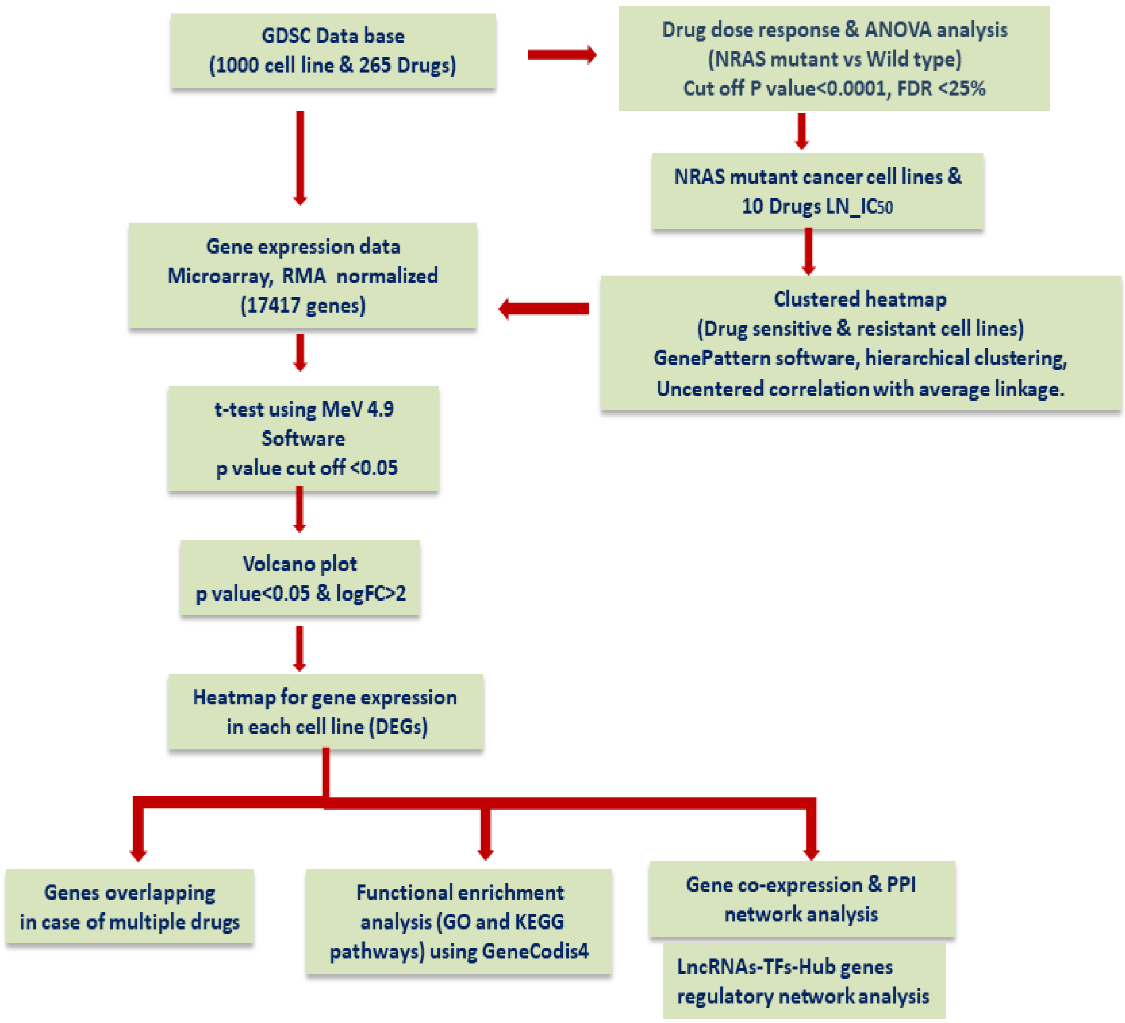

Figure S3a: Schematic overview depicting sequential methods used in our study.

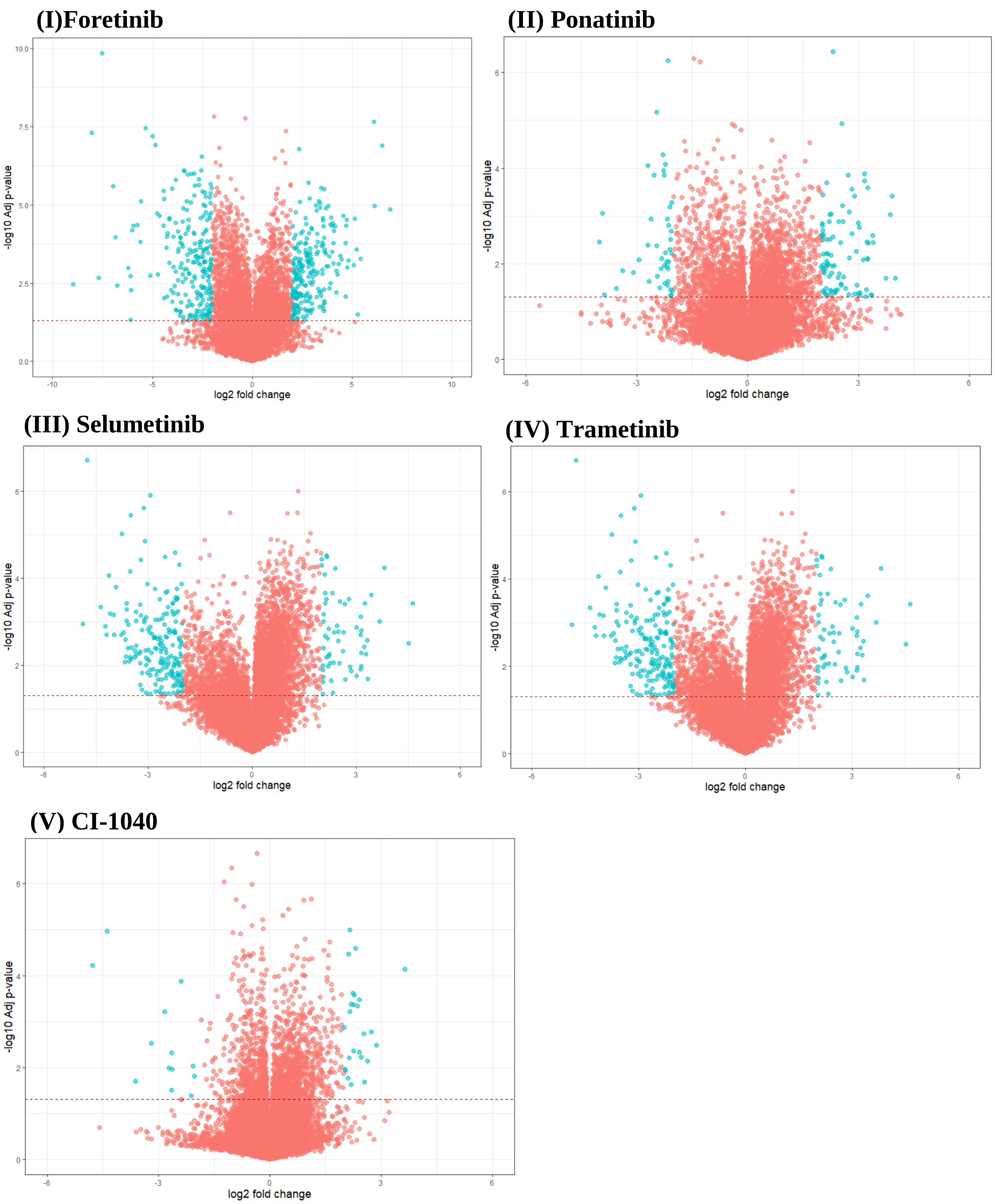

Figure S3b: Volcano plot of significantly differentially expressed genes between drug-sensitive and –resistant cancer cell lines. (I-V) Dots (blue) at the top represent significantly differentially expressed genes, bottom dots (red) represent non-significant differentially expressed genes. The x-axis shows fold change in gene expression (magnitude of change, logFC > 2) and the y-axis (p-value) shows statistically significant genes (threshold p-value <0.05) for each drug.
