# Supplementary material for: MALAT1 as master regulator of biomarkers predictive of pan-cancer multi-drug resistance in the context of recalcitrant NRAS signaling pathway identified using systems-oriented approach
Source: Sci Rep. 2022 May 9;12:7540. doi: 10.1038/s41598-022-11214-8 (PMC9085754; doi:10.1038/s41598-022-11214-8)
Supplement: Supplementary file 4 — Supplementary Figure S4. [file 41598_2022_11214_MOESM4_ESM.pdf]

# Functional enrichment analysis: GO and KEGG pathway

## (I)Ponatinib

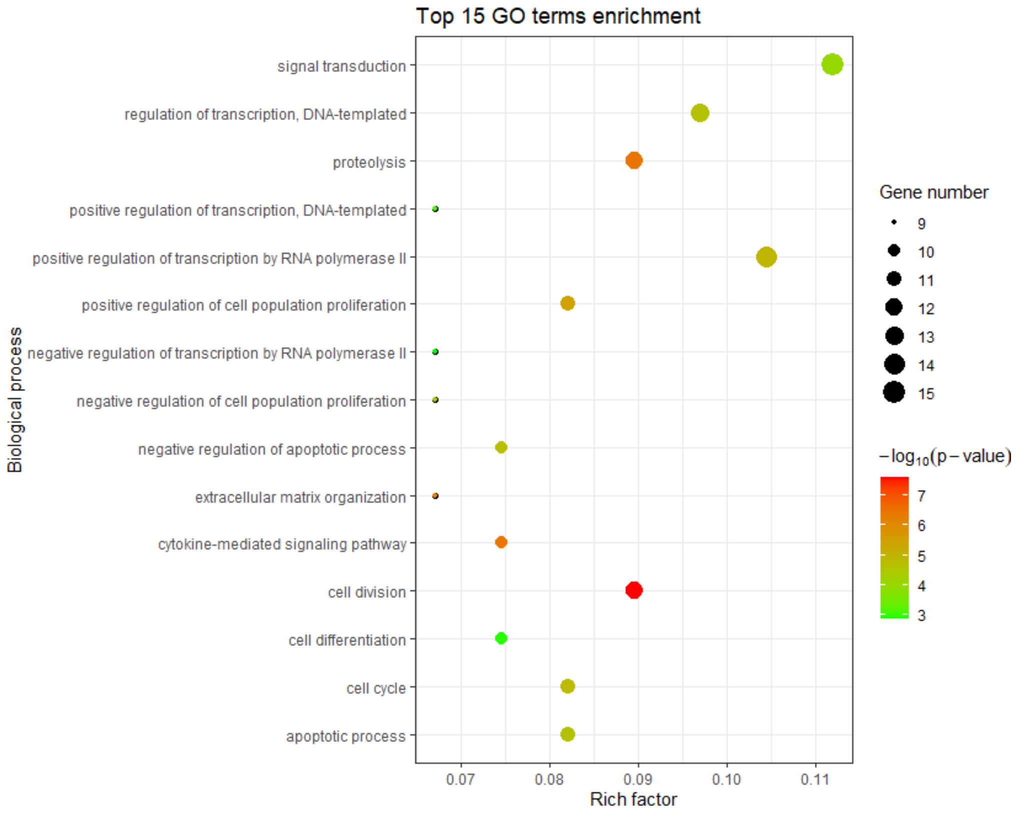


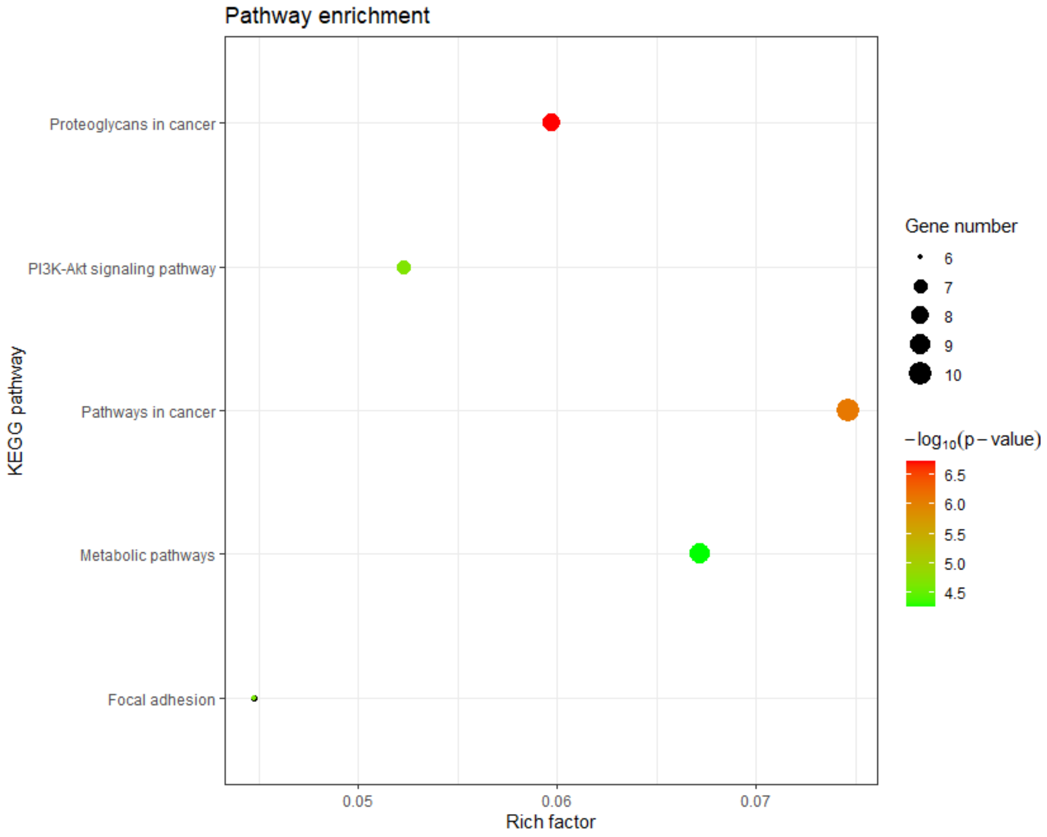

**(II)Foretinib**

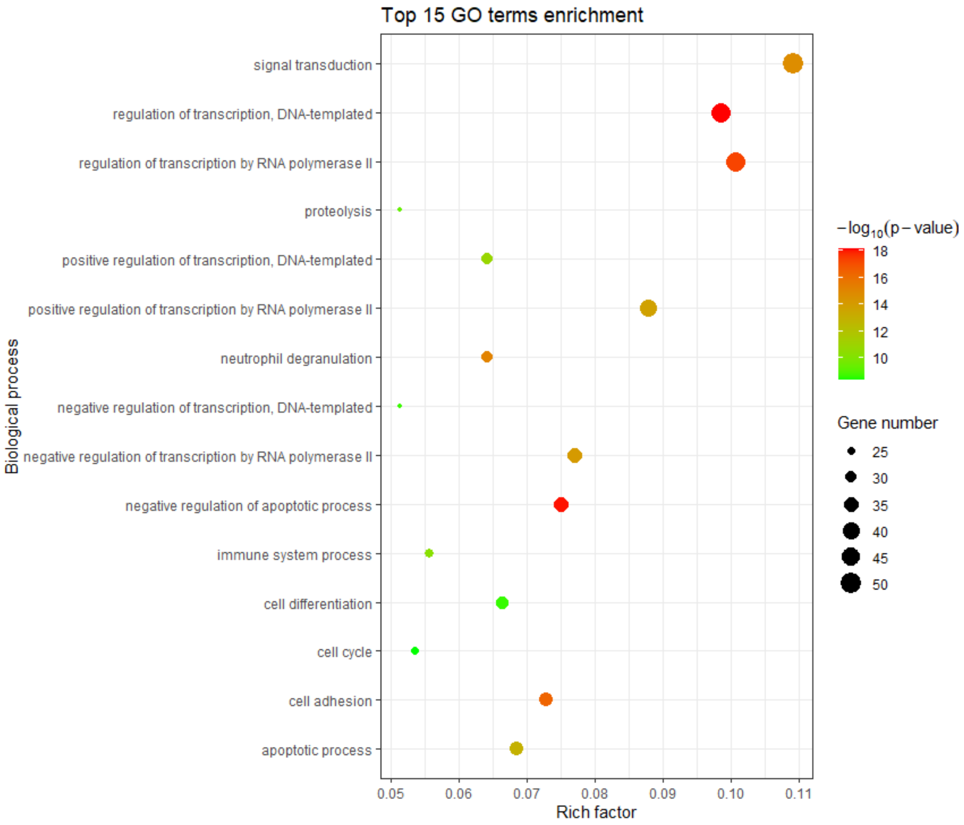


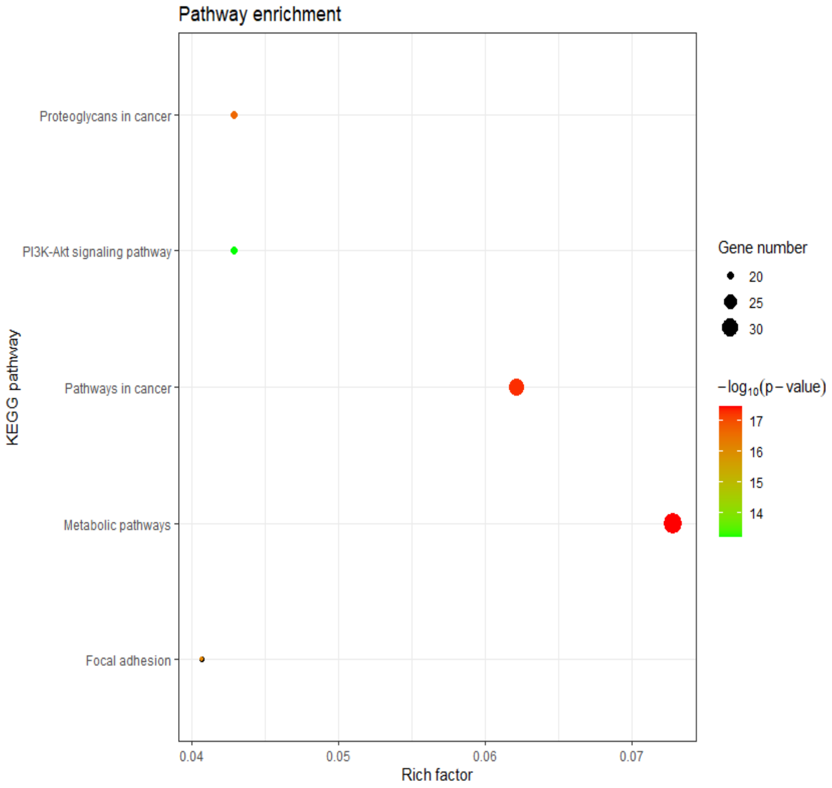

## (III) Selumetinib

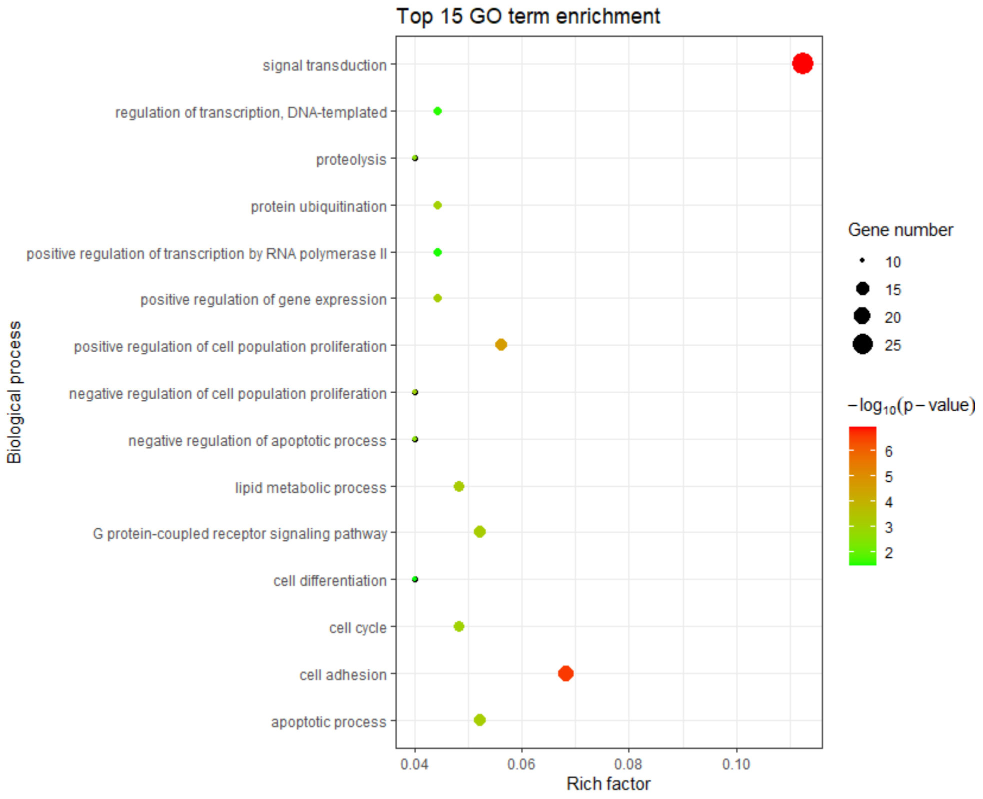


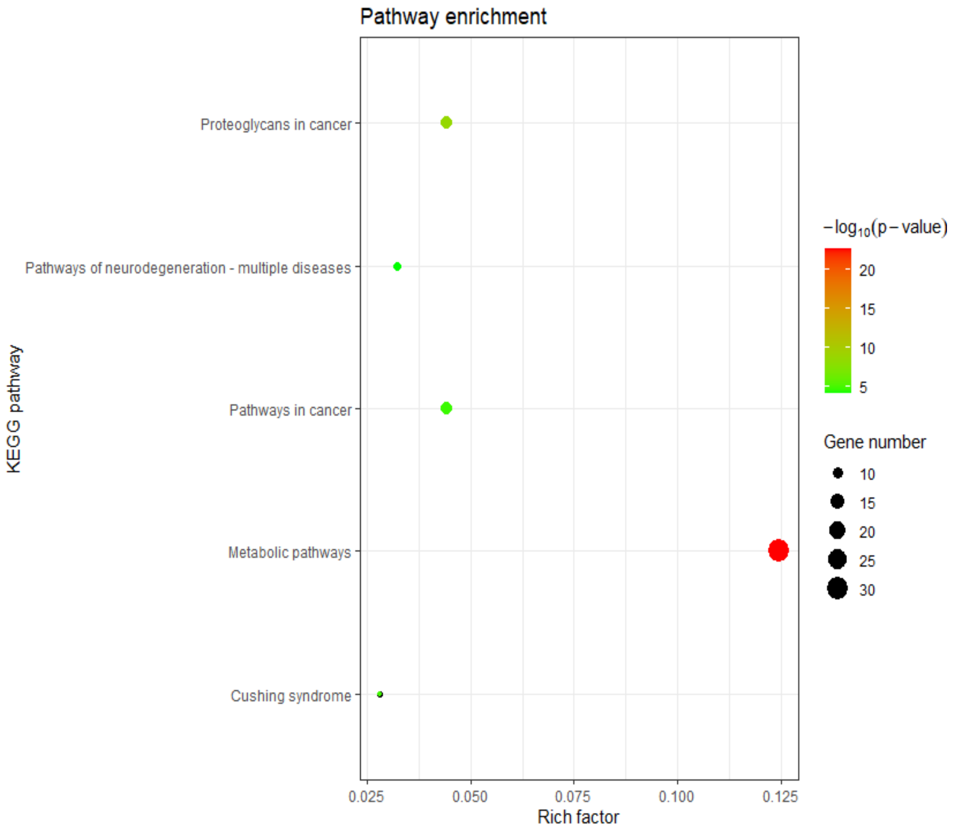

## (IV) Trametinib

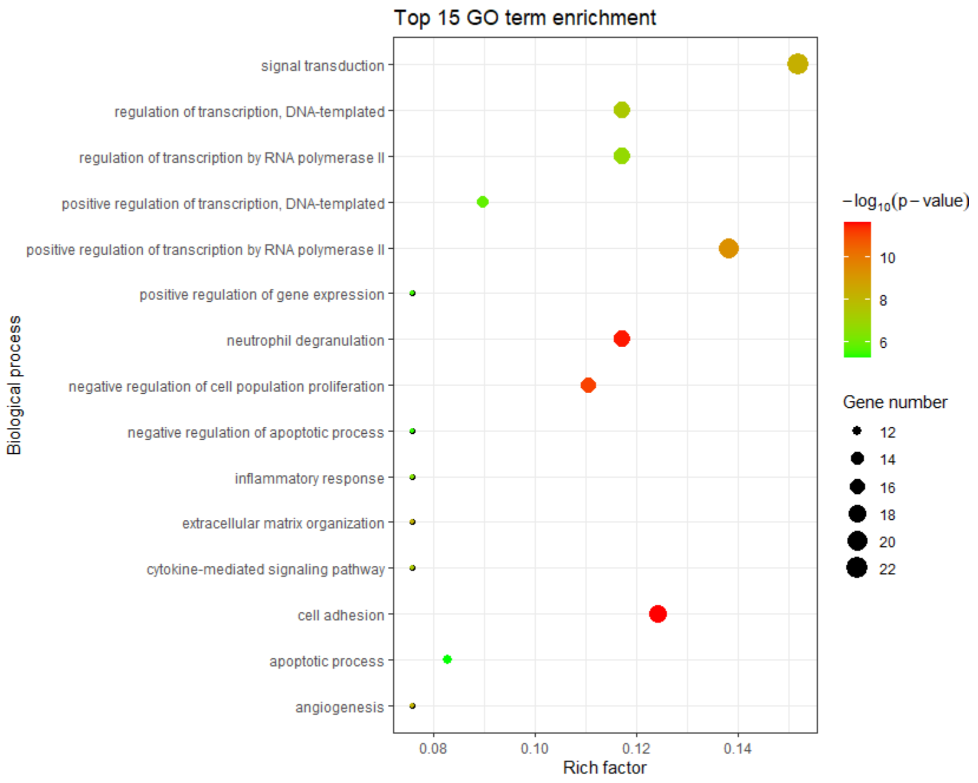


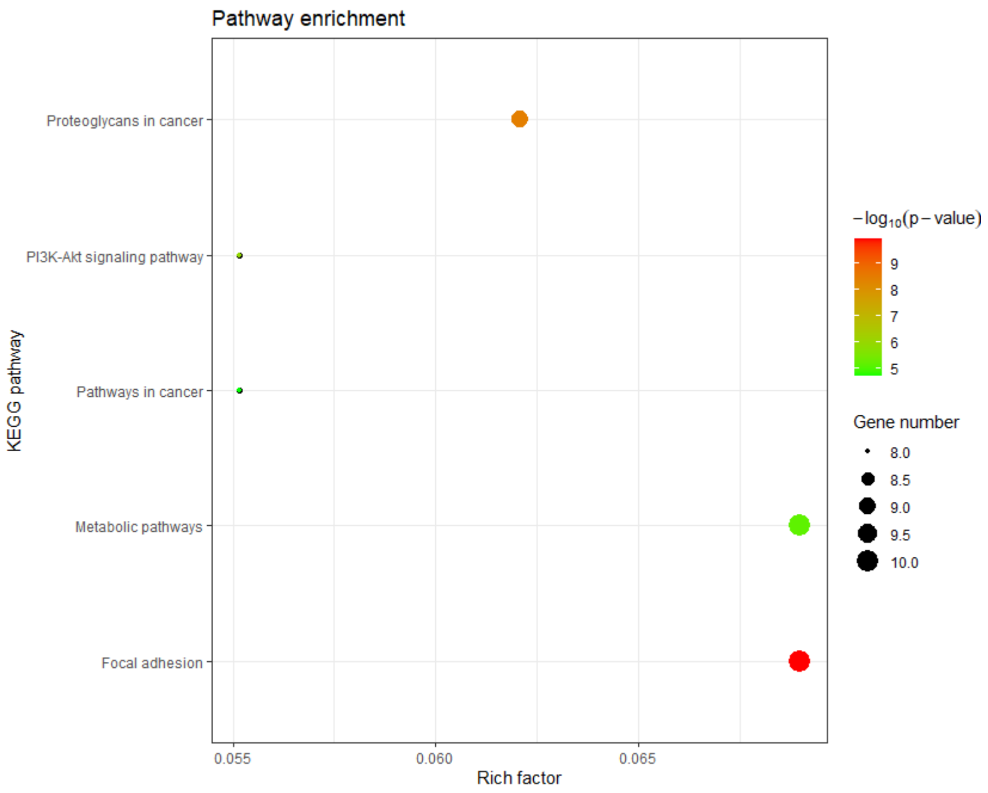

**(V)CI-1040**

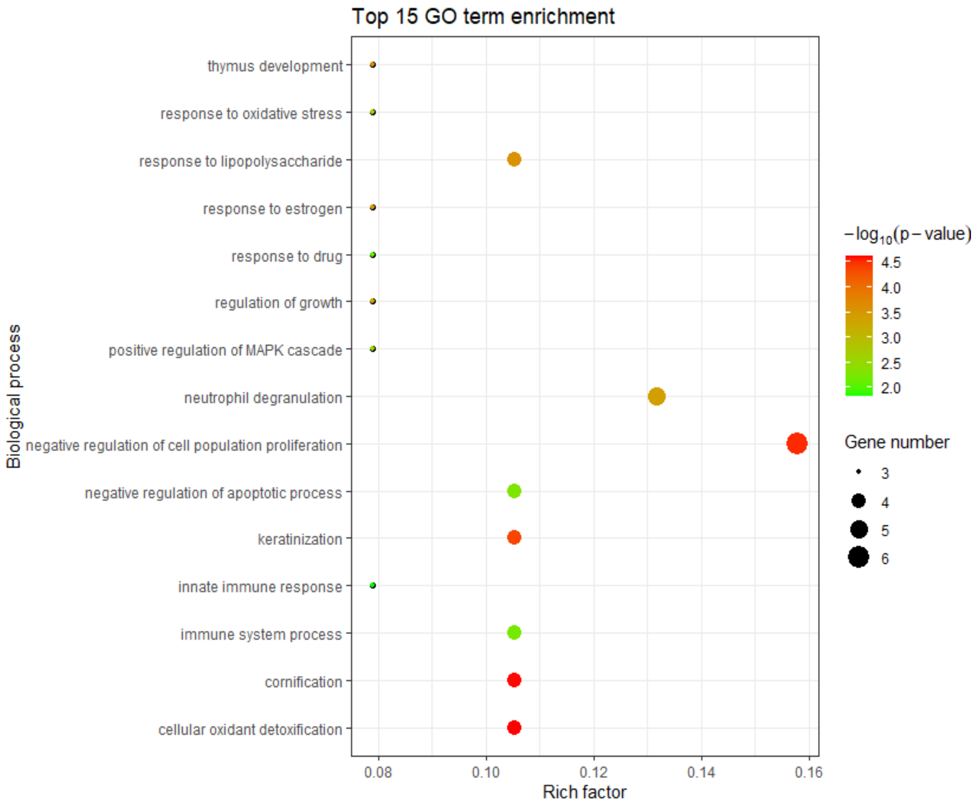


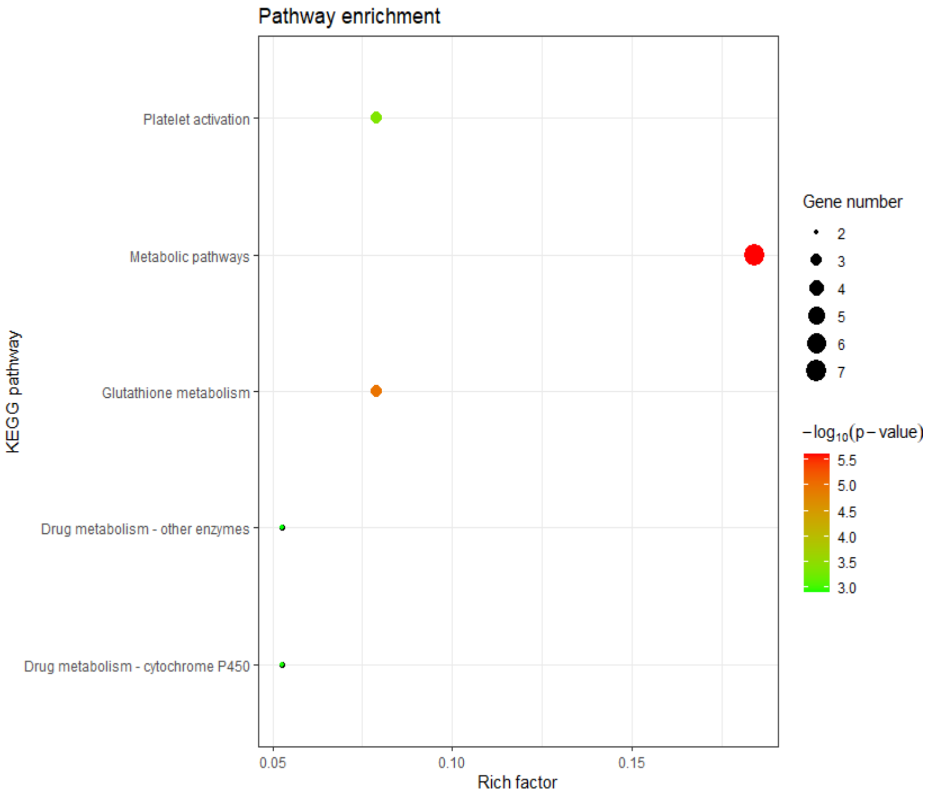


**Figure S4: Functional enrichment analysis: GO and KEGG pathway.** Bubble plot for Gene Ontology and KEGG pathway analysis of DEGs for five drugs, (I) Ponatinib, (II) Foretinib, (III) Selumetinib, (IV) Trametinib, (V) CI-1040. Top 15 GO terms enriched for

the biological process and top 5 KEGG pathway enrichment, at a default hypergeometric p-value of < or = 0.05.
