# Supplementary material for: MALAT1 as master regulator of biomarkers predictive of pan-cancer multi-drug resistance in the context of recalcitrant NRAS signaling pathway identified using systems-oriented approach
Source: Sci Rep. 2022 May 9;12:7540. doi: 10.1038/s41598-022-11214-8 (PMC9085754; doi:10.1038/s41598-022-11214-8)
Supplement: Supplementary file 5 — Supplementary Figure S5. [file 41598_2022_11214_MOESM5_ESM.pdf]

Gene co-expression network of DEGs

(I)Foretinib

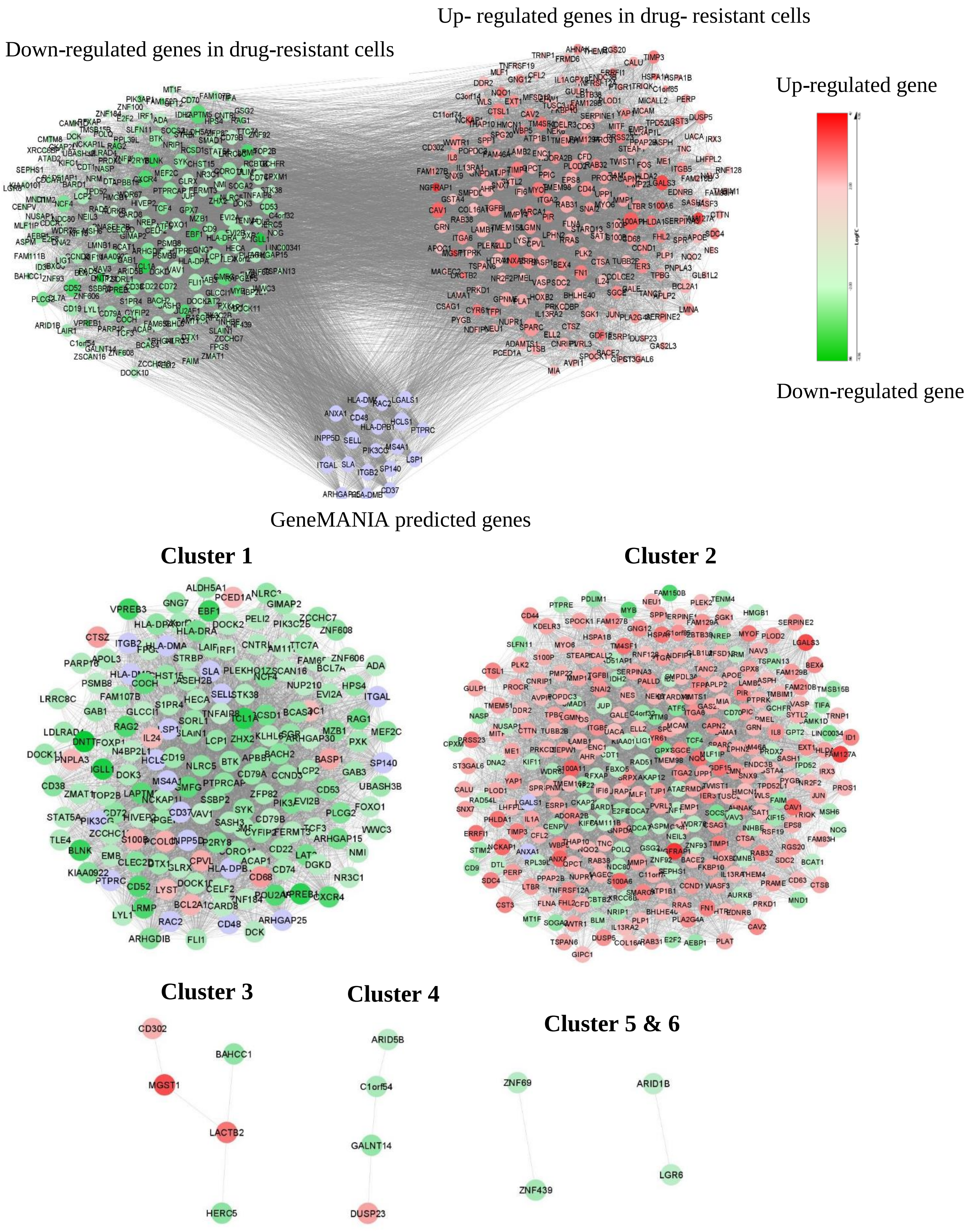

(II)Selumetinib

Down-regulated genes in drug-resistant cells

Up- regulated genes in drug- resistant cells

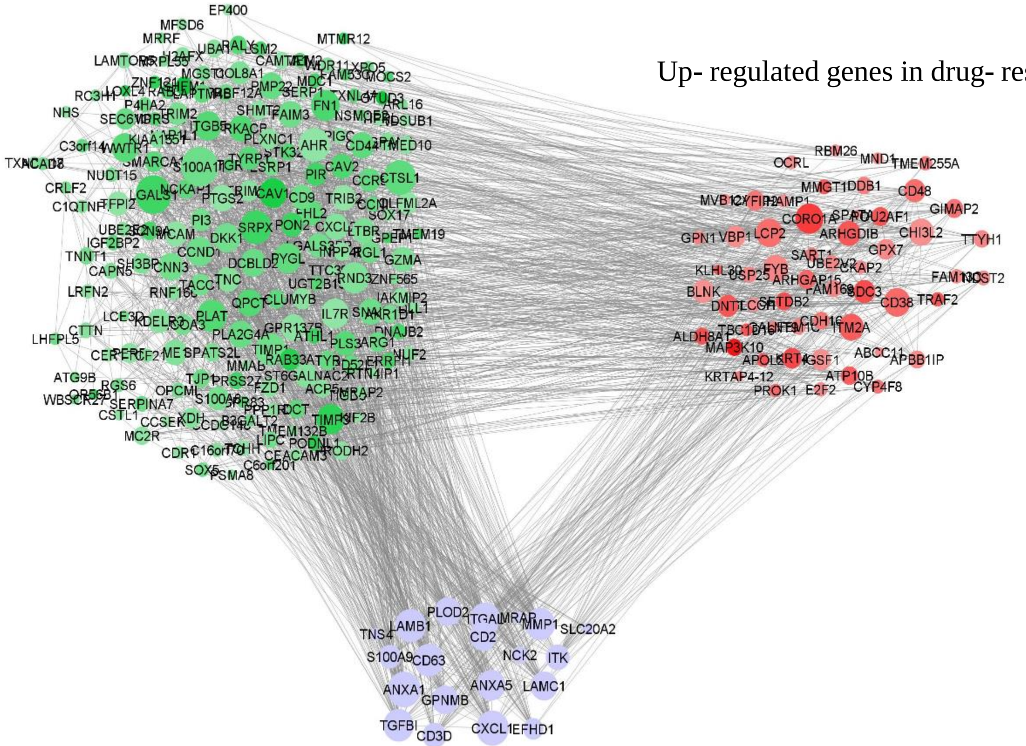

GeneMANIA predicted genes

Cluster 1

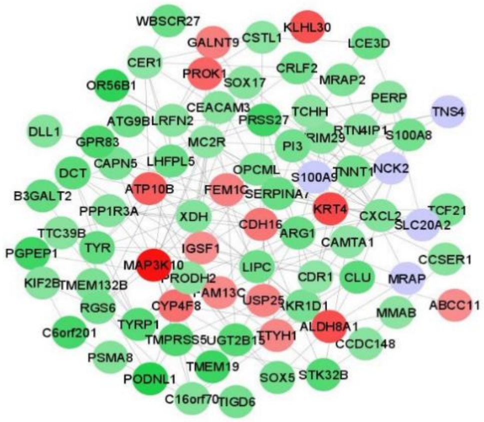

Cluster 2

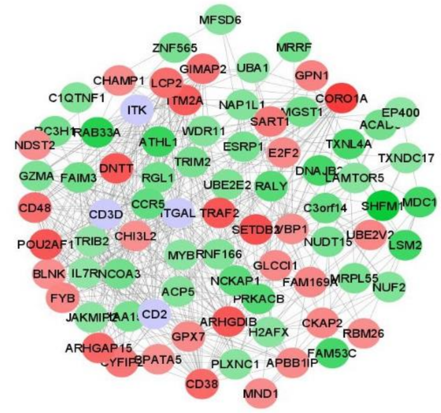

Cluster 3

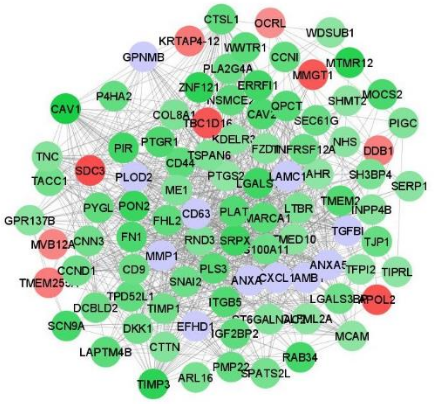

Cluster 4

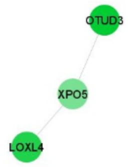

(III)Trametinib

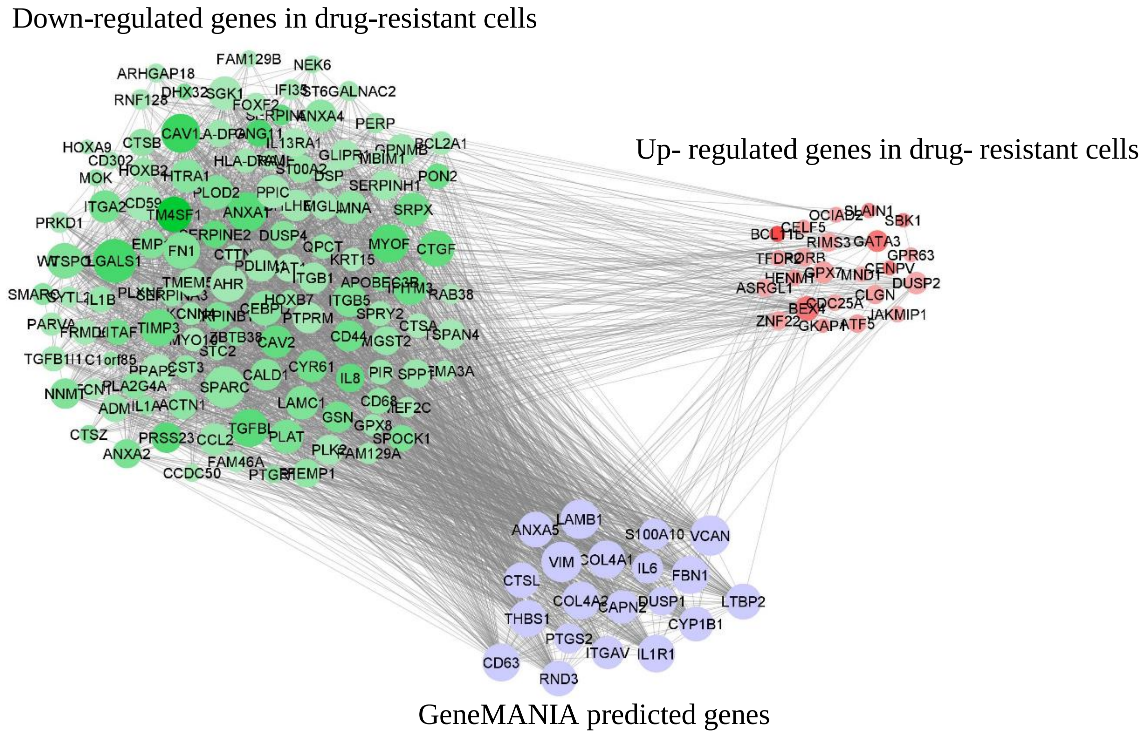

Cluster 1

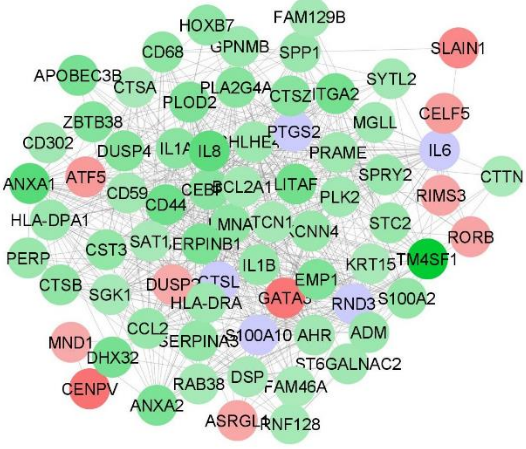

Cluster 2

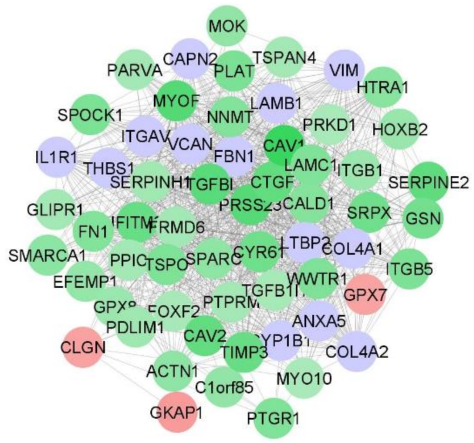

Cluster 3

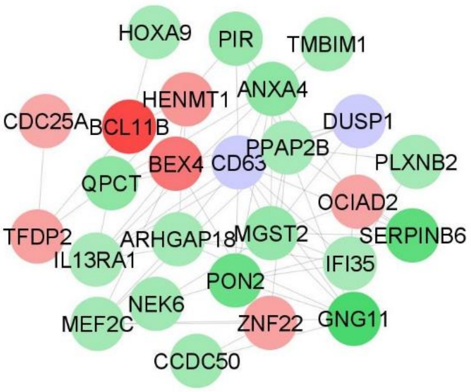

Cluster 4

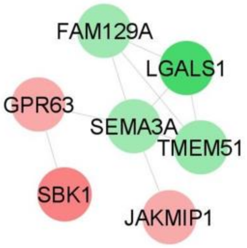

(IV)CI-1040

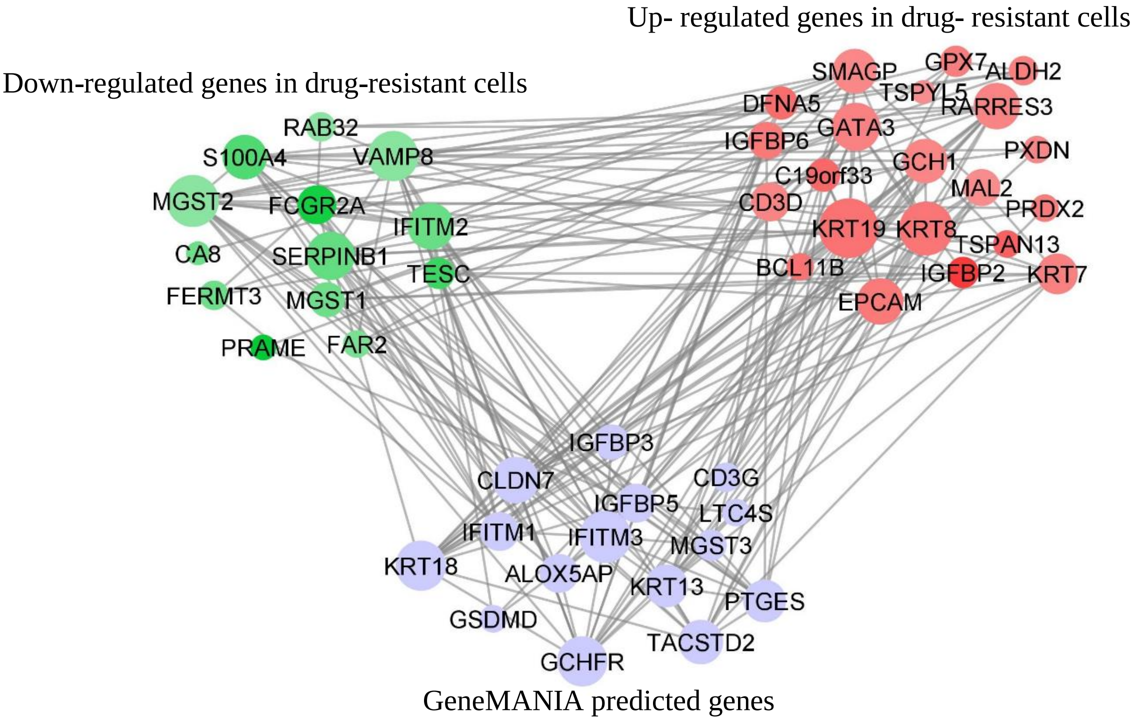

Cluster 1

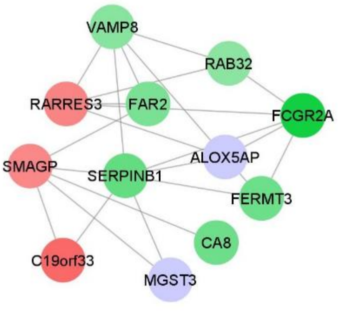

Cluster 2

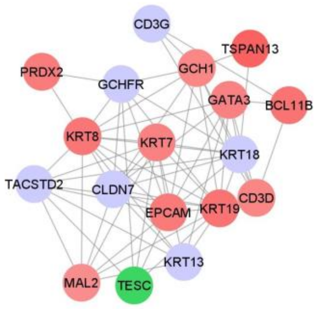

Cluster 3

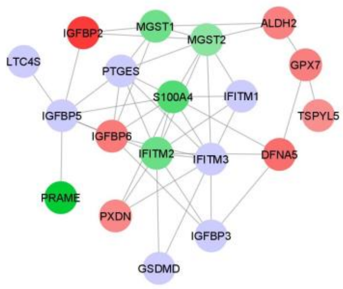

Figure S5: Gene co-expression network and clustered network. The co-expression network represents up- (red) and down-regulated genes (green) and GeneMANIA-predicted genes (blue), top hub genes detected by node degree (node size proportional to node degree) and clustered networks using Glay; (I) Foretinib, (II) Selumetinib, (III) Trametinib, (IV) CI-1040.
