# Supplementary material for: MALAT1 as master regulator of biomarkers predictive of pan-cancer multi-drug resistance in the context of recalcitrant NRAS signaling pathway identified using systems-oriented approach
Source: Sci Rep. 2022 May 9;12:7540. doi: 10.1038/s41598-022-11214-8 (PMC9085754; doi:10.1038/s41598-022-11214-8)
Supplement: Supplementary file 6 — Supplementary Figure S6. [file 41598_2022_11214_MOESM6_ESM.pdf]

Protein-protein interaction network of genes from co-expression network cluster and GO and KEGG analysis

(I) Foretinib

Cluster 2

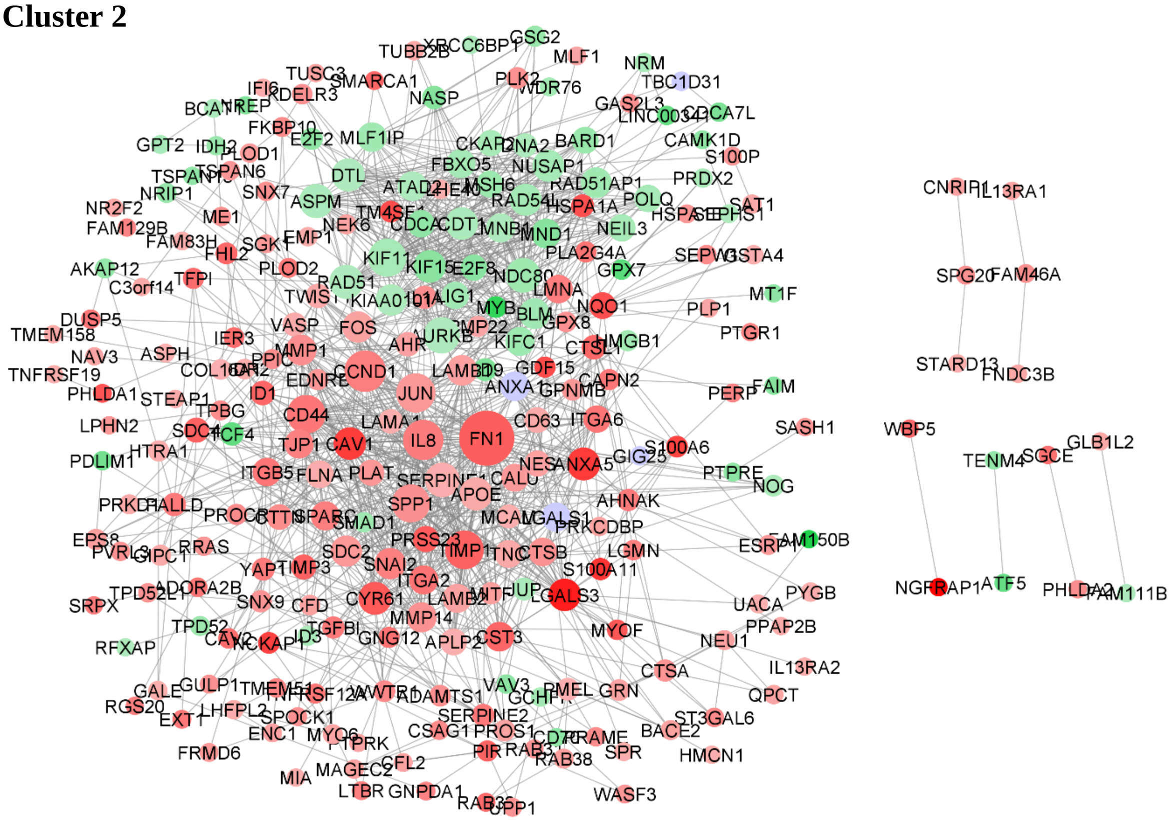

(II) Selumetinib

Cluster 2

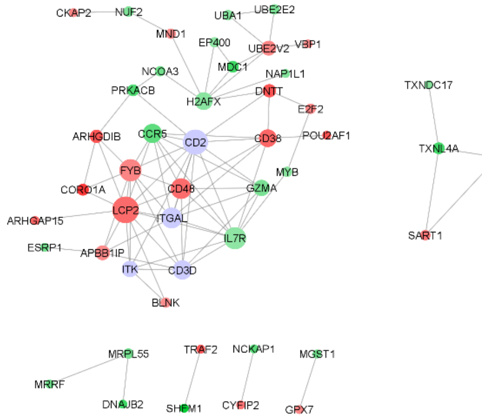

Cluster 3

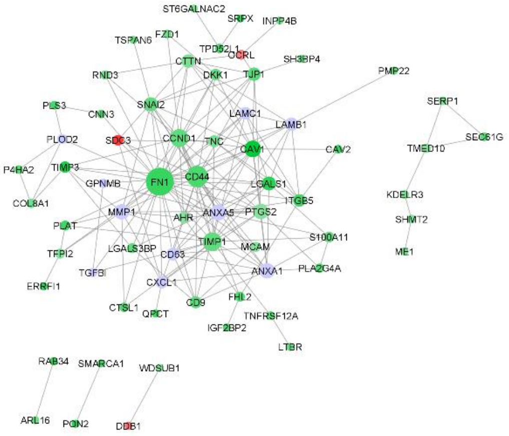

(III) Trametinib

Cluster 1

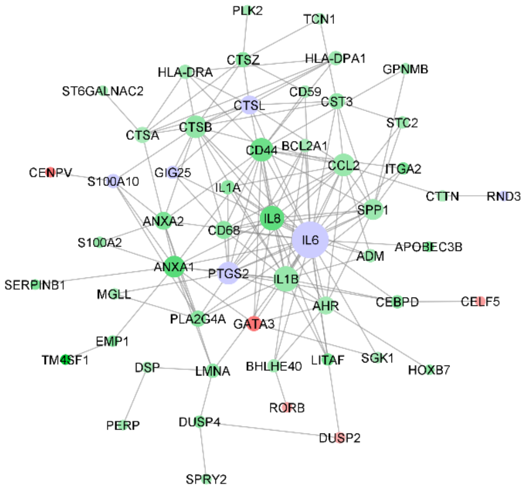

Cluster 2

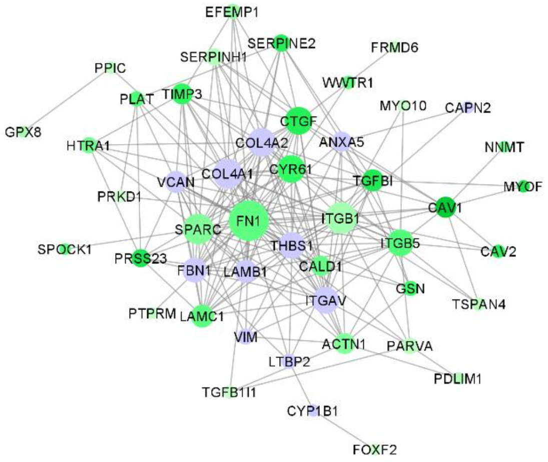

(IV) CI-1040

Cluster 2

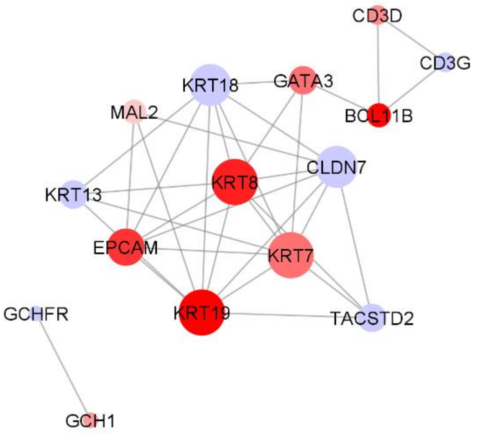

Figure S6a: Protein-protein interaction network of cluster. PPI network of genes from the co-expression network clusters for four drugs. (I) Foretinib, (II) Selumetinib, (III) Trametinib, (IV) CI-1040. Red nodes: upregulated protein-coding genes, Green nodes: down-regulated protein-coding genes, Blue nodes: GeneMANIA prediceted protein-coding genes.

(I) Ponatinib

Cluster 1

GO ID	Biological processes	genes_found	P-value
GO:0007049	cell cycle	13	1.29E-17
GO:0051301	cell division	10	3.33E-14
GO:0000278	mitotic cell cycle	5	2.45E-07
GO:0000086	G2/M transition of mitotic cell cycle	4	1.19E-05
GO:0008283	cell population proliferation	4	1.25E-05
GO:0006355	regulation of transcription, DNA-templated	4	0.00333

KEGG ID	KEGG Pathways	genes_found	P-value
hsa05169	Epstein-Barr virus infection	2	0.009102
hsa04110	Cell cycle	2	0.009265
hsa05203	Viral carcinogenesis	2	0.011292
hsa05200	Pathways in cancer	2	0.040845

Cluster 2

GO ID	Biological processes	genes_found	P-value
GO:0042438	melanin biosynthetic process	5	1.13E-12
GO:0043473	pigmentation	4	8.79E-08
GO:0007165	signal transduction	4	0.009021
GO:0032438	melanosome organization	3	5.74E-06
GO:0008284	positive regulation of cell population proliferation	3	0.006307
GO:0007399	nervous system development	3	0.006463
GO:0006583	melanin biosynthetic process from tyrosine	2	7.53E-06
GO:0006726	eye pigment biosynthetic process	2	1.81E-05
GO:0009637	response to blue light	2	5.02E-05
GO:0048066	developmental pigmentation	2	0.000236

KEGG ID	KEGG Pathways	genes_found	P-value
hsa04916	Melanogenesis	4	1.02E-07
hsa00350	Tyrosine metabolism	3	6.03E-07
hsa01100	Metabolic pathways	3	0.006029
hsa05014	Amyotrophic lateral sclerosis	2	0.007357
hsa05022	Pathways of neurodegeneration - multiple diseases	2	0.012748

Cluster 3

GO ID	Biological processes	genes_found	P-value
GO:0030198	extracellular matrix organization	12	8.64E-15
GO:0007165	signal transduction	12	2.75E-06
GO:0022617	extracellular matrix disassembly	8	9.83E-13
GO:0019221	cytokine-mediated signaling pathway	8	1.05E-07
GO:0006508	proteolysis	8	3.10E-06
GO:0007155	cell adhesion	7	5.26E-05
GO:0034097	response to cytokine	6	6.97E-09
GO:0030199	collagen fibril organization	6	8.84E-08
GO:0030335	positive regulation of cell migration	6	9.52E-06
GO:0008285	negative regulation of cell population proliferation	6	0.000155

KEGG ID	KEGG Pathways	genes_found	pval_adj
hsa05205	Proteoglycans in cancer	10	2.84E-14
hsa04510	Focal adhesion	6	2.13E-07
hsa05200	Pathways in cancer	6	2.24E-05
hsa04151	PI3K-Akt signaling pathway	5	3.35E-05
hsa04926	Relaxin signaling pathway	5	6.79E-07

(II)Foretinib

Cluster 2

GO ID	Biological processes	genes_found	P-value
GO:0007165	signal transduction	31	1.69E-10
GO:0045944	positive regulation of transcription by RNA polymerase II	27	3.83E-11
GO:0007155	cell adhesion	26	3.12E-16
GO:0043066	negative regulation of apoptotic process	25	2.95E-16
GO:0000122	negative regulation of transcription by RNA polymerase II	21	3.44E-09
GO:0030154	cell differentiation	21	5.00E-08
GO:0030198	extracellular matrix organization	20	1.00E-16
GO:0006915	apoptotic process	20	1.80E-09
GO:0043312	neutrophil degranulation	19	5.06E-11
GO:0007049	cell cycle	19	4.20E-09

KEGG ID	KEGG Pathways	genes_found	P-value
hsa01100	Metabolic pathways	23	1.17E-14
hsa05200	Pathways in cancer	21	8.42E-16
hsa04510	Focal adhesion	17	3.50E-18
hsa05205	Proteoglycans in cancer	17	7.89E-18
hsa04151	PI3K-Akt signaling pathway	13	4.99E-10

(III)Selumetinib

Cluster 2

GO ID	Biological processes	genes_found	P-value
GO:0007165	signal transduction	9	0.000456
GO:0007049	cell cycle	6	0.000472
GO:0006915	apoptotic process	6	0.000513
GO:0006955	immune response	5	0.000501
GO:0045944	positive regulation of transcription by RNA polymerase II	5	0.012815
GO:0006303	double-strand break repair via nonhomologous end joining	4	5.16E-05
GO:0050852	T cell receptor signaling pathway	4	0.000594
GO:0007166	cell surface receptor signaling pathway	4	0.00126
GO:0008380	RNA splicing	4	0.001276
GO:0006397	mRNA processing	4	0.002943

KEGG ID	KEGG Pathways	genes_found	P-value
hsa05200	Pathways in cancer	6	2.08E-05
hsa04640	Hematopoietic cell lineage	5	2.63E-07
hsa05169	Epstein-Barr virus infection	5	4.75E-06
hsa04015	Rap1 signaling pathway	4	0.00014
hsa05163	Human cytomegalovirus infection	4	0.000146

Cluster 3

GO ID	Biological processes	genes_found	P-value
GO:0007155	cell adhesion	15	5.35E-14
GO:0007165	signal transduction	11	4.47E-05
GO:0030198	extracellular matrix organization	9	3.04E-09
GO:0008285	negative regulation of cell population proliferation	9	2.41E-07
GO:0043066	negative regulation of apoptotic process	9	6.06E-07
GO:0019221	cytokine-mediated signaling pathway	8	2.66E-07
GO:0002576	platelet degranulation	7	2.31E-08
GO:0044267	cellular protein metabolic process	7	6.32E-08
GO:0016477	cell migration	7	1.13E-06
GO:0001525	angiogenesis	7	1.14E-06

KEGG ID	KEGG Pathways	genes_found	P-value
hsa05205	Proteoglycans in cancer	11	1.26E-15
hsa04510	Focal adhesion	8	1.32E-10
hsa05165	Human papillomavirus infection	8	9.11E-10
hsa01100	Metabolic pathways	8	3.35E-06
hsa05200	Pathways in cancer	7	3.40E-06

(IV)Trametinib

Cluster 1

GO ID	Biological processes	genes_found	P-value
GO:0006954	inflammatory response	12	1.21E-12
GO:0043312	neutrophil degranulation	12	3.93E-12
GO:0019221	cytokine-mediated signaling pathway	10	5.47E-11
GO:0008285	negative regulation of cell population proliferation	10	1.42E-09
GO:0007165	signal transduction	10	3.84E-05
GO:0006955	immune response	8	2.27E-07
GO:0045944	positive regulation of transcription by RNA polymerase II	8	0.000133
GO:0071222	cellular response to lipopolysaccharide	7	3.40E-08
GO:0010628	positive regulation of gene expression	7	2.22E-05
GO:0043066	negative regulation of apoptotic process	7	2.24E-05

KEGG ID	KEGG pathways	genes_found	P-value
hsa05323	Rheumatoid arthritis	8	4.83E-14
hsa04640	Hematopoietic cell lineage	8	6.32E-14
hsa05164	Influenza A	7	1.92E-10
hsa05321	Inflammatory bowel disease	6	1.73E-10
hsa04142	Lysosome	6	1.75E-10

Cluster 2

GO ID	Biological processes	genes_found	P-value
GO:0007155	cell adhesion	16	1.07E-18
GO:0030198	extracellular matrix organization	15	2.97E-22
GO:0001525	angiogenesis	11	1.87E-14
GO:0044267	cellular protein metabolic process	7	4.02E-09
GO:0016477	cell migration	7	7.38E-08
GO:0043687	post-translational protein modification	7	1.79E-07
GO:0007165	signal transduction	7	0.001849
GO:0007229	integrin-mediated signaling pathway	6	2.15E-08
GO:0002576	platelet degranulation	6	4.34E-08
GO:0030335	positive regulation of cell migration	6	1.99E-06

KEGG ID	KEGG pathways	genes_found	P-value
hsa04510	Focal adhesion	14	5.36E-25
hsa04512	ECM-receptor interaction	9	7.62E-19
hsa05165	Human papillomavirus infection	9	3.73E-13
hsa04151	PI3K-Akt signaling pathway	9	1.63E-12
hsa05205	Proteoglycans in cancer	8	2.26E-12

(V)CI-1040
Cluster 2

GO ID	Biological process	genes_found	P-value
GO:0070268	cornification	5	1.14E-09
GO:0031424	keratinization	5	1.16E-09
GO:0065003	protein-containing complex assembly	3	0.000124
GO:0050852	T cell receptor signaling pathway	3	0.000185
GO:0043066	negative regulation of apoptotic process	3	0.001989
GO:0002376	immune system process	3	0.002215
GO:0045944	positive regulation of transcription by RNA polymerase II	3	0.005357

KEGG ID	KEGG pathways	genes_found	P-value
hsa05150	Staphylococcus aureus infection	3	2.54E-06
hsa04659	Th17 cell differentiation	3	5.89E-06
hsa04658	Th1 and Th2 cell differentiation	3	6.04E-06
hsa04915	Estrogen signaling pathway	3	6.73E-06

Figure 6b: GO and KEGG pathways analysis of proteins from PPI network. Gene ontology and KEGG pathway analysis using GeneCodis4 for five drugs. (I) Ponatinib, (II) Foretinib, (III) Selumetinib, (IV) Trametinib, (V) CI-1040.
