# Supplementary material for: MALAT1 as master regulator of biomarkers predictive of pan-cancer multi-drug resistance in the context of recalcitrant NRAS signaling pathway identified using systems-oriented approach
Source: Sci Rep. 2022 May 9;12:7540. doi: 10.1038/s41598-022-11214-8 (PMC9085754; doi:10.1038/s41598-022-11214-8)
Supplement: Supplementary file 7 — Supplementary Table S1. [file 41598_2022_11214_MOESM7_ESM.pdf]

Sr. no	Drug	Drug Target	Effect size	P-value	FDR%	No. of altered cell lines
1	PD0325901	MEK1, MEK2	-0.901	1.51E-10	0.000167	54
2	RDEA119	MEK1, MEK2	-0.916	4.75E-10	0.000392	54
3	Trametinib	MEK1, MEK2	-0.75	5.36E-07	0.17	55
4	Selumetinib	MEK1, MEK2	-0.798	8.74E-07	0.267	57
5	TL-1-85	TAK	-0.0932	7.33E-06	1.34	58
6	RDEA119	MEK1, MEK2	-0.797	8.08E-06	1.42	53
7	CI-1040	MEK1, MEK2	-0.853	8.77E-06	1.49	56
8	NG-25	TAK1, MAP4K2	-0.00937	9.73E-06	1.64	58
9	Ponatinib	ABL, PDGFRA, VEGFR2, FGFR1, SRC, TIE2, FLT3	0.101	2.74E-05	3.43	58
10	Cabozantinib	VEGFR, MET, RET, KIT, FLT1, FLT3, FLT4, TIE2,AXL	0.168	0.000132	9.14	58
11	Foretinib		0.189	0.000261	14	57
12	PLX-4720	BRAF	-0.041	0.000297	14.9	55

Table S1:- Compounds with their targets showing effect size, and number of altered cell lines against a target specific drug. (https://www.cancerrxgene.org)
