# Supplementary material for: MALAT1 as master regulator of biomarkers predictive of pan-cancer multi-drug resistance in the context of recalcitrant NRAS signaling pathway identified using systems-oriented approach
Source: Sci Rep. 2022 May 9;12:7540. doi: 10.1038/s41598-022-11214-8 (PMC9085754; doi:10.1038/s41598-022-11214-8)
Supplement: Supplementary file 8 — Supplementary Table S2. [file 41598_2022_11214_MOESM8_ESM.pdf]

TCGA Classification	Cancer cell lines
ALL	P12-ICHIKAWA, DND-41, KE-37, MOLT-4, PF-382, HAL-01.
BLCA	HT-1197, KU-19-19, BFTC-905,
DLBC	OCI-LY-19
LIHC	C3A
LUAD	NCI-H2347, NCI-H2087.
LUSC	HCC-15
LAML	THP-1, ME-1, KY821, OCI-AML3, HL-60, KMOE-2
MB	ONS-76
MM	L-363, JJN-3
NB	GOTO
SCLC	SW1271
THCA	ASH-3
SKCM	IPC-298, LB373-MEL-D, GAK, MEL-JUSO, LB2518-MEL, CP66-MEL, SK-MEL-2, MZ2-MEL
Unclassified	MFH-ino, SJSA-1, HT-1080, TYK-nu, NCI-H2135, HD-MY-Z, 697

Table S2a: Names of 41 cell lines studied corresponding to cancer types as identified from TCGA.

Sr No.	Drug name		No. of drug-resistant cell lines	No. of drug-sensitive cell lines
**1**	Selumetinib		16	10
**2**	CI-1040		21	3
**3**	PD-0325901		1	31
**4**	Trametinib		5	24
**5**	TL-1-85		39	0
**6**	NG-25		36	0
**7**	Cabozantinib		40	0
**8**	PLX4720		41	0
**9**	Foretinib		12	3
**10**	Ponatinib		14	4

Table S2b: Number of drug-sensitive and -resistant cancer cell lines identified by normalized IC_{50} score for 10 drugs.

Sr no.	Drugs	Up-regulated genes	Down-regulated genes	Total no. of DEGs

		in drug-resistant cells	in drug-resistant cells	genes
**1**	Selumetinib	60	189	249
**4**	CI-1040	25	13	38
**5**	Trametinib	23	122	145
**6**	Ponatinib	90	44	134
**7**	Foretinib	236	231	467

Table S2c: Number of significantly DEGs (up-and down-regulated) in drug-sensitive and resistant pan-cancer cell lines.
