# Supplementary material for: MALAT1 as master regulator of biomarkers predictive of pan-cancer multi-drug resistance in the context of recalcitrant NRAS signaling pathway identified using systems-oriented approach
Source: Sci Rep. 2022 May 9;12:7540. doi: 10.1038/s41598-022-11214-8 (PMC9085754; doi:10.1038/s41598-022-11214-8)
Supplement: Supplementary file 9 — Supplementary Table S3. [file 41598_2022_11214_MOESM9_ESM.pdf]

(a) Ponatinib

Sr No.	Genes	Degree	Sr No.	Genes	Degree
1	*SPARC*	63	18	*TWIST1*	35
2	*SRPX*	59	19	*PROCR*	35
3	*PTPRM*	51	20	*CNRIP1*	35
4	*PIR*	48	21	*BCL2A1*	34
5	*EDNRB*	47	22	*SERPINE2*	33
6	*FN1*	45	23	*TYR*	32
7	*VEGFC*	44	24	*S100B*	32
8	*TMEM158*	42	25	*CTSL1*	32
9	*PLP1*	42	26	*CD44*	32
10	*HTRA1*	41	27	*SLC6A15*	32
11	*SNAI2*	41	28	*IL13RA2*	31
12	*DDR2*	40	29	*LMNB1*	31
13	*TIMP3*	39	30	*PLK2*	31
14	*SGK1*	39	31	*STC1*	31
15	*TIMP1*	39	32	*PMEL*	30
16	*MLANA*	38	33	*MYB*	30
17	*MMP1*	38	34	*MMP14*	30

(b) Foretinib

**S No.**	**Gene name**	**Degree**	**S No.**	**Gene name**	**Degree**
1	*LAPTM5*	145	27	*CAV1*	111
2	*SPARC*	139	28	*SYK*	111
3	*S100A11*	139	29	*LRMP*	111
4	*EVI2B*	138	30	*LAMB1*	108
5	*CORO1A*	132	31	*TNFAIP8*	106
6	*GMFG*	132	32	*AEBP1*	106
7	*CD52*	129	33	*LYL1*	106
8	*PTPRCAP*	128	34	*EPS8*	105
9	*CXCR4*	128	35	*CD63*	105
10	*EVI2A*	126	36	*CD38*	105
11	*FLI1*	125	37	*SRPX*	105
12	*TGFBI*	124	38	*CD44*	105
13	*RAB31*	124	39	*LGALS3*	105
14	*BTK*	120	40	*LCP1*	105
15	*CD53*	120	41	*MYOF*	105
16	*VAV1*	120	42	*FN1*	104
17	*NCF4*	120	43	*FAM65B*	104
18	*SASH3*	120	44	*GLRX*	103
19	*ARHGDIB*	119	45	*CD19*	103
20	*AHR*	117	46	*CCND3*	103
21	*MEF2C*	117	47	*NCKAP1L*	103
22	*LCP2*	115	48	*WWTR1*	102
23	*TIMP1*	113	49	*CD79B*	100
24	CTSL1	113	50	KIAA0922	100

25	*HLA-DPA1*	112	51	*CD72*	100
26	*CD79A*	112	52	*PLEKHO1*	100

(c) Selumetinib

S No.	Gene name	Degree	S No.	Gene name	Degree
1	*S100A11*	60	25	*FYB*	35
2	*LGALS1*	58	26	*CHI3L2*	35
3	*CTSL1*	50	27	*FAIM3*	34
4	*SRPX*	48	28	*GPR137B*	34
5	*AHR*	48	29	*PMP22*	34
6	*TIMP1*	46	30	*ITM2A*	34
7	*PLAT*	45	31	*PTGS2*	34
8	*RND3*	43	32	*PRKACB*	33
9	*FN1*	42	33	*SNAI2*	33
10	*PYGL*	41	34	*CD44*	33
11	*IL7R*	41	35	*CXCL2*	33
12	*CORO1A*	41	36	*ME1*	32
13	*TIMP3*	41	37	*TRIB2*	32
14	*CAV1*	40	38	*FHL2*	31
15	*DKK1*	39	39	*MCAM*	31
16	*CCND1*	39	40	*TNC*	31
17	*ITGB5*	39	41	*ARHGDIB*	31
18	*WWTR1*	39	42	*GZMA*	31
19	*CD38*	38	43	*LTBR*	31
20	*LCP2*	38	44	*PLS3*	30
21	*CNN3*	36	45	*LGALS3BP*	30
22	*DCBLD2*	36	46	*CAV2*	30
23	*PLA2G4A*	35	47	*ST6GALNAC2*	30
24	*QPCT*	35	48	*CD9*	30

(d) Trametinib

S No.	Gene name	Degree	S No.	Gene name	Degree
1	*LGALS1*	87	19	*ITGB5*	58
2	*SPARC*	80	20	*SRPX*	57
3	*ANXA1*	74	21	*LAMC1*	57
4	*CAV1*	73	22	*PPAP2B*	56
5	*MYOF*	71	23	*PPIC*	56
6	*TGFBI*	70	24	*HTRA1*	56
7	*TIMP3*	67	25	*LMNA*	55
8	*AHR*	66	26	*ANXA4*	54
9	*FN1*	66	27	*ITGA2*	54
10	*CD59*	64	28	*CAV2*	54
11	*CTGF*	64	29	*CCL2*	53
12	*WWTR1*	63	30	*PDLIM1*	53
13	*TM4SF1*	62	31	*CYR61*	52
14	*CEBPD*	60	32	*BHLHE40*	51
15	*PLOD2*	59	33	*CALD1*	51

16	*IFITM3*	59	34	*CD44*	50
17	*PLAT*	58	35	*PTPRM*	50
18	*SGK1*	58			

(e) CI-1040

**S No.**	**Gene Name**	**Degree**
1	*KRT19*	20
2	*KRT8*	17
3	*MGST2*	16
4	*VAMP8*	15
5	*GATA3*	14
6	*SERPINB1*	14
7	*RARRES3*	13
8	*IFITM2*	13
9	*EPCAM*	13
10	*S100A4*	12
11	*GCH1*	12
12	*SMAGP*	12
13	*KRT7*	10

Table S3: List of hub genes identified from co-expression network for five drugs. (a) Ponatinib, b) Foretinib, c) Selumetinib, d) Trametinib, e) CI-1040
