# Supplementary material for: MALAT1 as master regulator of biomarkers predictive of pan-cancer multi-drug resistance in the context of recalcitrant NRAS signaling pathway identified using systems-oriented approach
Source: Sci Rep. 2022 May 9;12:7540. doi: 10.1038/s41598-022-11214-8 (PMC9085754; doi:10.1038/s41598-022-11214-8)
Supplement: Supplementary file 10 — Supplementary Table S4. [file 41598_2022_11214_MOESM10_ESM.pdf]

(a) Ponatinib

Cluster 1

Sr No.	Name	Degree
1	NDC80	20
2	AURKB	20
3	KIF15	19
4	FBXO5	18
5	CDT1	18
6	ATAD2	17
7	NCAPH	17
8	MND1	16
9	CDCA7	15
10	E2F8	15

Cluster 2

Sr No.	Name	Degree
1	DCT	8
2	TYR	8
3	TYRP1	7
4	PMEL	7
5	MLANA	6
6	PRAME	5
7	EDNRB	5

Cluster 2

Sr No.	Name	Degree
1	FN1	24
2	CD44	15
3	MMP1	15
4	TIMP1	15
5	MMP14	14
6	SPARC	13
7	SNAI2	12
8	VEGFC	10
9	TIMP3	10
10	FOS	7

(b)Foretinib

Cluster 2

**Name**	**Degree**
FN1	66
CCND1	42
IL8	40
JUN	38
TIMP1	37
CD44	37
SPP1	36
KIF11	36
AURKB	33
SERPINE1	30

(c)Selumetinib

Cluster 2

Name	Degree
LCP2	13
FYB	10
IL7R	10
CD48	9
CCR5	8
CD38	7
H2AFX	7
GZMA	6
APBB1IP	5
UBE2V2	5

Cluster 3

Name	Degree
FN1	31
CD44	21
TIMP1	17
CCND1	16
CAV1	14
PTGS2	12
SNAI2	10
LGALS1	10
TJP1	9
CTTN	9

(d) Trametinib

Cluster 1

name	Degree
IL1B	17
IL8	16
CD44	15
CCL2	14
CTSB	14
ANXA1	13
SPP1	12
AHR	9
ANXA2	9
CD68	9

Cluster 2

Name	Degree
FN1	31
ITGB1	22
SPARC	21
CYR61	18
CTGF	18
ITGB5	17
LAMC1	13
TGFBI	12
ACTN1	11
CAV1	11

(e)CI-1040

Cluster 2

Name	Degree
KRT7	8
KRT8	8
KRT19	8
EPCAM	6

Table S4: List of Identified top hub proteins from PPI network of clusters. (a) Ponatinib, b) Foretinib, c) Selumetinib, d) Trametinib, e) CI-1040.
