# Supplementary material for: MALAT1 as master regulator of biomarkers predictive of pan-cancer multi-drug resistance in the context of recalcitrant NRAS signaling pathway identified using systems-oriented approach
Source: Sci Rep. 2022 May 9;12:7540. doi: 10.1038/s41598-022-11214-8 (PMC9085754; doi:10.1038/s41598-022-11214-8)
Supplement: Supplementary file 11 — Supplementary Table S5. [file 41598_2022_11214_MOESM11_ESM.pdf]

Drug name	Cluster	Number of hub protein nodes from PPI network	Number of nodes in PPI network
Ponatinib	Cluster 1	10	25
	Cluster 2	7	22
	Cluster 3	10	59
Foretinib	Cluster 2	10	254
Selumetinib	Cluster 2	10	47
	Cluster 3	10	67
Trametinib	Cluster 1	10	54
	Cluster 2	10	47
CI-1040	Cluster 2	4	15

Table S5: Number of top hub protein nodes identified from each PPI network clusters in case of all five drugs.
