# Supplementary material for: MALAT1 as master regulator of biomarkers predictive of pan-cancer multi-drug resistance in the context of recalcitrant NRAS signaling pathway identified using systems-oriented approach
Source: Sci Rep. 2022 May 9;12:7540. doi: 10.1038/s41598-022-11214-8 (PMC9085754; doi:10.1038/s41598-022-11214-8)
Supplement: Supplementary file 12 — Supplementary Table S6. [file 41598_2022_11214_MOESM12_ESM.docx]

| Genes | Chromosomal location | Gene ID |
| --- | --- | --- |
| *LncRNA-MALAT1* | 11q13.1 | 378938 |
| *FN1* | 2q35 | 2335 |
| *CD44* | 11p13 | 960 |
| *TIMP1* | Xp11.3 | 7076 |
| *SNAI2* | 8q11.2 | 6591 |
| *SPARC* | 5q33.1 | 6678 |

**Table S6a**: Chromosomal location of selected coding genes and lncRNA.

| LncRNA | Hub genes | Interaction site |
| --- | --- | --- |
| *MALAT1* | *FN1*  *CD44*  *TIMP1*  *SPARC*  *SNAI2* | 5’UTR  3’UTR  CDS  3’UTR  CDS |

**Table S6b:** Predicted LncRNA-mRNA interaction site of coding hub genes.
